# The ion channels of endomembranes

**DOI:** 10.1152/physrev.00025.2023

**Published:** 2024-03-07

**Authors:** Meiqin Hu, Xinghua Feng, Qiang Liu, Siyu Liu, Fangqian Huang, Haoxing Xu

**Affiliations:** ^1^Department of Neurology and Department of Cardiology, The Second Affiliated Hospital of Zhejiang University School of Medicine, Hangzhou, People’s Republic of China; ^2^New Cornerstone Science Laboratory, Liangzhu Laboratory and School of Basic Medical Sciences, Zhejiang University, Hangzhou, People’s Republic of China; ^3^Department of Molecular, Cellular, and Developmental Biology, University of Michigan, Ann Arbor, Michigan, United States

**Keywords:** IP_3_R, RyR, TMEM175, TPC2, TRPML1

## Abstract

The endomembrane system consists of organellar membranes in the biosynthetic pathway [endoplasmic reticulum (ER), Golgi apparatus, and secretory vesicles] as well as those in the degradative pathway (early endosomes, macropinosomes, phagosomes, autophagosomes, late endosomes, and lysosomes). These endomembrane organelles/vesicles work together to synthesize, modify, package, transport, and degrade proteins, carbohydrates, and lipids, regulating the balance between cellular anabolism and catabolism. Large ion concentration gradients exist across endomembranes: Ca^2+^ gradients for most endomembrane organelles and H^+^ gradients for the acidic compartments. Ion (Na^+^, K^+^, H^+^, Ca^2+^, and Cl^−^) channels on the organellar membranes control ion flux in response to cellular cues, allowing rapid informational exchange between the cytosol and organelle lumen. Recent advances in organelle proteomics, organellar electrophysiology, and luminal and juxtaorganellar ion imaging have led to molecular identification and functional characterization of about two dozen endomembrane ion channels. For example, whereas IP3R1–3 channels mediate Ca^2+^ release from the ER in response to neurotransmitter and hormone stimulation, TRPML1–3 and TMEM175 channels mediate lysosomal Ca^2+^ and H^+^ release, respectively, in response to nutritional and trafficking cues. This review aims to summarize the current understanding of these endomembrane channels, with a focus on their subcellular localizations, ion permeation properties, gating mechanisms, cell biological functions, and disease relevance.

## Graphical abstract

**Figure:**
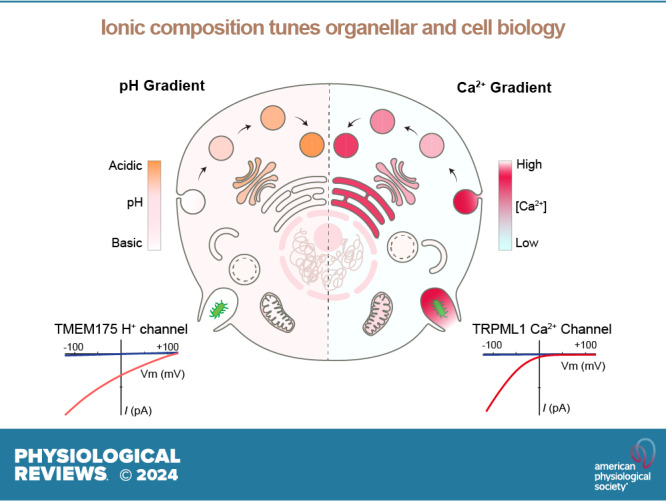

CLINICAL HIGHLIGHTS Intracellular compartments of the cell host various endomembrane ion channels and transporters that are responsible for the regulation of luminal ionic composition, organellar membrane potential, vesicular trafficking, lipid and protein synthesis, macromolecular degradation, and signal transduction.

In particular, organellar pH and Ca^2+^ gradients, produced and coordinated by organellar Ca^2+^/H^+^ transporters and channels, are essential for organellar and cellular functions that include muscle contraction, hormone secretion, transmitter release, endocytosis, exocytosis, autophagy, nutrient sensing, lysosomal degradation, catabolite export, and organelle biogenesis. Dysregulation of organellar pH or Ca^2+^ homeostasis and signaling may cause diverse human pathologies including lysosome storage diseases, neurodegenerative diseases, metabolic diseases, infectious diseases, and cancer.

The activities of endomembrane ion channels are regulated by cellular cues within the biosynthetic secretory and degradative pathways. Small-molecule modulators of endomembrane ion channels may mimic endogenous cellular cues to tune organellar and cellular functions, providing new strategies for disease intervention.

## 1. INTRODUCTION

### 1.1. Ion Channels in the Endomembrane: Fast Messaging Between the Lumen and the Cytosol

**FIGURE 1.**
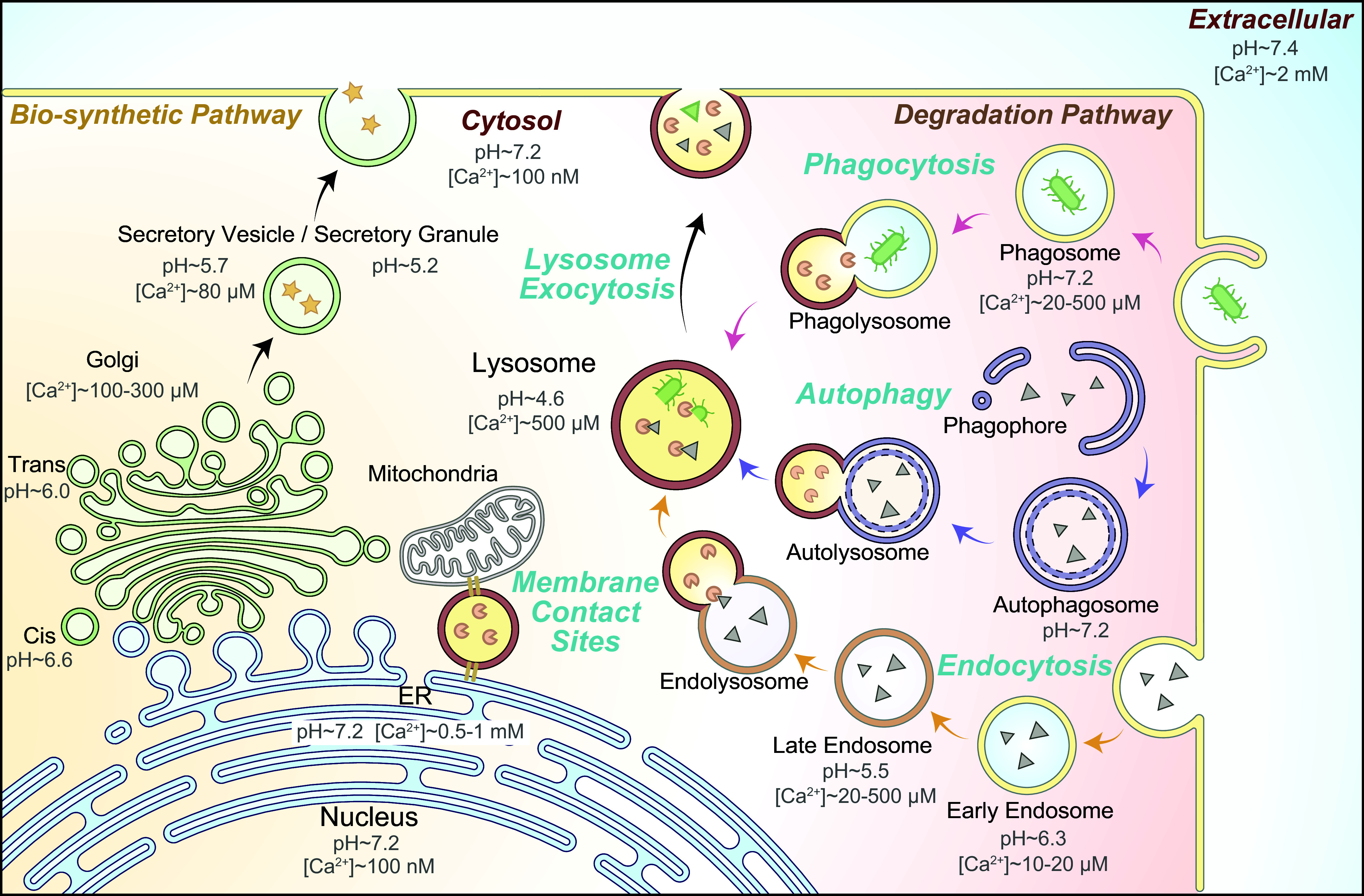
The endomembrane system: biosynthetic vs. degradative pathways. The endomembrane organelles of the biosynthetic secretory pathway consist of the nucleus, the endoplasmic reticulum (ER), the Golgi apparatus [*cis*-Golgi and *trans*-Golgi network (TGN)], and secretory vesicles/granules; the endomembrane organelles of the degradative pathway consist of early endosomes, phagosomes, autophagosomes, late endosomes, and lysosomes. Each endomembrane organelle has a unique ionic composition, including Ca^2+^ and H^+^. Whereas the cytosolic Ca^2+^ level is as low as 100 nM, endomembrane organelles are intracellular Ca^2+^ stores, with the luminal Ca^2+^ concentrations ranging from 10 to 1,000 μM. Acidic organelles use vacuolar-type (V-)ATPases to produce transmembrane pH gradients, and lysosomes represent the most acidic organelle in the cell, with a pH of 4.5–5.0. In the biosynthetic pathway, proteins and lipids are synthetized in the ER, modified in the Golgi apparatus, and delivered to the destination places, e.g., the plasma membrane through secretory vesicles. In the degradative pathway, cargo macromolecules are delivered to lysosomes through endocytosis, phagocytosis, and autophagy. In the late stages of endocytosis and autophagy, endosomes, phagosomes, and autophagosomes fuse with lysosomes to form endolysosomes, phagolysosomes, and autolysosomes, respectively, in which cargo degradation mediated by lysosomal hydrolases takes places in the lumen. Ions, proteins, lipids, and other cargoes can be transported between organelles through both vesicular membrane trafficking and nonvesicular membrane contact sites (MCSs). In response to cellular cues, organellar Ca^2+^ and H^+^ may regulate organelle membrane sorting, trafficking (fusion and fission), and formation of MCSs.

Unlike prokaryotic cells, eukaryotic cells are filled with intracellular membranes that serve at least two roles: compartmentalization and signaling (1–5). The organelles in the endomembrane system of a mammalian cell can be categorized on the basis of their functions into two distinct pathways: the biosynthetic secretory pathway, which consists of the nucleus, the endoplasmic reticulum (ER), the Golgi apparatus, and secretory vesicles/granules, and the degradative pathway, which consists of early endosomes, macropinosomes, phagosomes, autophagosomes, late endosomes, and lysosomes (2, 6) (FIGURE 1). The plasma membrane serves as either the destination or initiation station, respectively, in these two complementary pathways. Whereas the biosynthetic pathway synthesizes complex macromolecules such as proteins from simpler molecules such as amino acids, the degradation pathway plays an opposite role by breaking down complex and larger macromolecules into simpler building-block molecules (5, 7–9). Although catabolism and anabolism are opposite cellular processes, the organelles and vesicles in the biosynthetic and degradative pathways do communicate and exchange materials with each other, e.g., via vesicular membrane trafficking and nonvesicular organelle-organelle membrane contact (6, 7, 10) (FIGURE 1). To achieve a balance and homeostasis of the cell, cross talk mechanisms between and within the pathways must exist on endomembrane organelles (2, 11). There exist various sensing mechanisms on the organellar membranes from both branches, and nutrient-dependent signaling molecules, e.g., mammalian target of rapamycin (mTOR), may regulate both processes, catabolism and anabolism (12–15). mTOR is regulated by the levels of metabolites, e.g., amino acids, which are the source materials for biosynthesis but the product materials from degradation (12, 16–19). Hence, common signaling molecules may regulate both ER and lysosomal functions, e.g., the rate of macromolecular biosynthesis versus the rate of macromolecular degradation (12, 18, 19). For instance, nutrient starvation may stimulate lysosomal degradation while halting biosynthesis (20, 21). To coordinate the regulation, informational exchange must occur constantly between the biosynthetic organelles and the cytosol, between the degradative organelles and the cytosol, and between the biosynthetic and degradative organelles (13, 22, 23). Notably, there exist large (>3-fold, up to 10,000-fold) ion gradients across the endomembranes, with Ca^2+^ gradients for all endomembrane organelles except the nucleus and with Na^+^, K^+^, H^+^, and Cl^−^ gradients for most endomembrane organelles (2, 24–27) (see FIGURE 1). Luminal pH decreases gradually as the secretory organelles maturate anterogradely en route to the destination, i.e., the plasma membrane, and when the degradative organelles maturate retrogradely en route to the destination, i.e., the lysosome (28–30) (see FIGURE 1). Hence, each endomembrane organelle may engage a distinct set of ion flux mechanisms for both lumen-to-cytosol and cytosol-to-lumen signal transduction.

Ion flux mechanisms are extensively studied across the plasma membrane, yet >80% of ion transport processes occur across intracellular membranes (2). Ion flux across intracellular membranes such as the ER, Golgi apparatus, endosomes, and lysosomes is difficult to investigate and remained poorly understood until recent technical advances in both organellar patch-clamp electrophysiology and organelle-targeted fluorescent ion imaging (2, 24, 25, 31–35). In the past two decades, about two dozen new organellar channels on the endomembrane were molecularly identified and electrophysiologically characterized; molecular identification has made it possible to study the physiological functions of organellar channels with genetic and pharmacological manipulations (2, 36–39). Organellar channels are now known to regulate numerous cellular processes, ranging from catabolism to anabolism and from cell proliferation to cell death: organellar ion homeostasis, signal transduction, membrane fusion and fission, organelle-organelle membrane contact, macromolecular synthesis, macromolecular degradation, nutrient sensing, organelle membrane repair, organelle reformation, and organelle biogenesis (2, 13, 25, 27, 36–38, 40).

A major function of endomembrane ion flux is to regulate intracellular Ca^2+^ signaling, the most common signal transduction mechanism in cells (1, 3, 13, 25). Intracellular Ca^2+^ signaling plays important roles in the regulation of signal transduction, membrane trafficking, organelle homeostasis, and organelle-organelle membrane contact (13, 24, 41, 42). Virtually all endomembrane organelles in the biosynthetic and degradative pathways are considered to be intracellular Ca^2+^ stores, with luminal Ca^2+^ concentration ([Ca^2+^]_Lumen_) ranging from tens of micromolar (μM) to millimolar (mM), 100- to 10,000-fold higher than the level of resting cytosolic Ca^2+^ ([Ca^2+^]_Cytosol_, ∼100 nM) (2, 3, 24, 26, 27, 39) (see FIGURE 1). Many Ca^2+^ channels and transporters are enriched in intracellular organelles (2). For example, inositol (1,4,5)-trisphosphate (IP_3_) receptors (IP3R1–3) are Ca^2+^-permeable channels in the ER, the primary Ca^2+^ store in the cell (1, 3, 37, 39). IP3Rs are the essential signal transduction player in the phospholipase C (PLC) pathway that is stimulated by numerous neurotransmitters and hormones (1, 3, 37, 40). Likewise, mucolipin TRP (TRPML1–3) channels in the late endosomes and lysosomes (LELs), in response to various cellular cues, regulate membrane trafficking, fusion and fission, exocytosis, organelle membrane contact, autophagosome formation, and lysosome biogenesis (13, 41, 43, 44). Additionally, intracellular transport of other ions such as H^+^, Na^+^, K^+^, and Cl^−^ regulates both luminal ionic composition and organellar membrane potential (Δψ), the latter of which is known to affect organellar Ca^2+^ release/uptake indirectly (13, 45, 46). For instance, whereas ER K^+^ channels may affect both the uptake and release of Ca^2+^ across ER membranes, lysosomal K^+^ channels regulate lysosomal Ca^2+^ release and store refilling (2, 45–49). Nevertheless, these ions also regulate other organellar functions independent of Ca^2+^. For example, whereas endolysosomal Na^+^ channels and transporters may regulate metabolite transport, organellar content condensation, and membrane fission (15, 36, 50, 51), high intralysosomal Cl^−^ is required for the activities of some lysosomal hydrolases (52–55).

**FIGURE 2.**
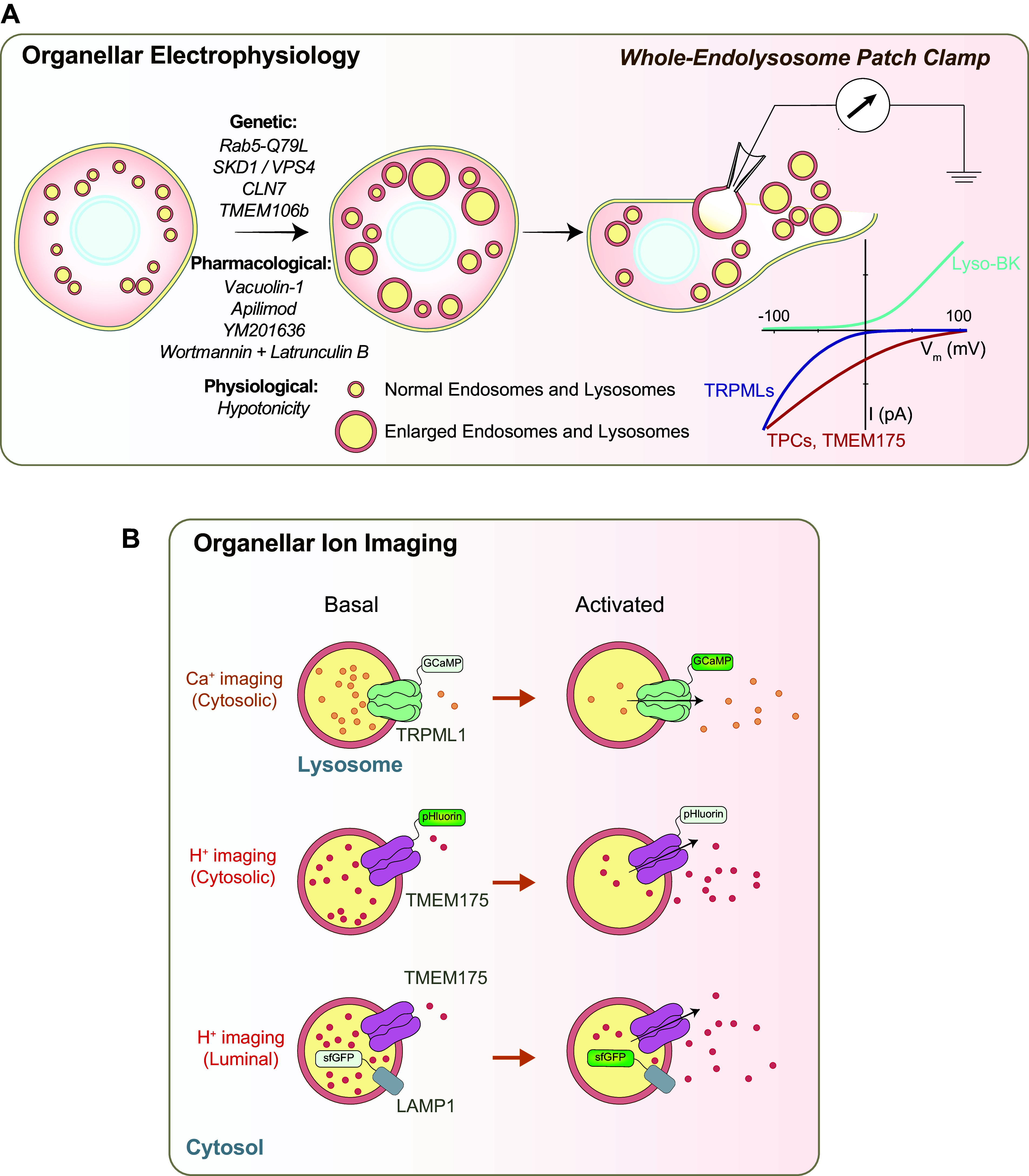
Methods to study endomembrane ion channels. *A*: ion channels of endomembranes can be studied with organellar electrophysiology. In whole endolysosome patch-clamp recordings, after glass electrodes are used to break the cell endolysosomes that are genetically, pharmacologically, or physiologically enlarged are manually isolated. The cytosolic side (bath) is connected to the ground electrode, and the luminal side (pipette) is filled with the recording pipette solution. In whole endolysosome recording, cation efflux from the luminal side to the cytosolic side is defined as inward currents. Whereas mucolipin TRP channels (TRPMLs) are Ca^2+^-permeable channels in the lysosomes, two-pore channels (TPCs) and TMEM175 are lysosomal Na^+^-selective and H^+^-selective channels, respectively. *I*, current; *V*_m_, membrane potential. *B*: fluorescent imaging with organelle-targeted genetically encoded ion indicators can be used to study ion flux across organellar membranes. For example, GCaMPs tagged to the cytoplasmic side of lysosomal TRPML1 channels can detect lysosomal Ca^2+^ release upon stimulation. Whereas juxtalysosomal H^+^ release can be measured by tagging pHluorin (p*K*_a_ ∼6.9) to the cytoplasmic side of lysosomal TMEM175 channels, luminal pH can be monitored by tagging superfolder green fluorescent protein (sfGFP) (p*K*_a_ ∼5.6) to the luminal side of a lysosomal membrane protein such as LAMP1.

**FIGURE 3.**
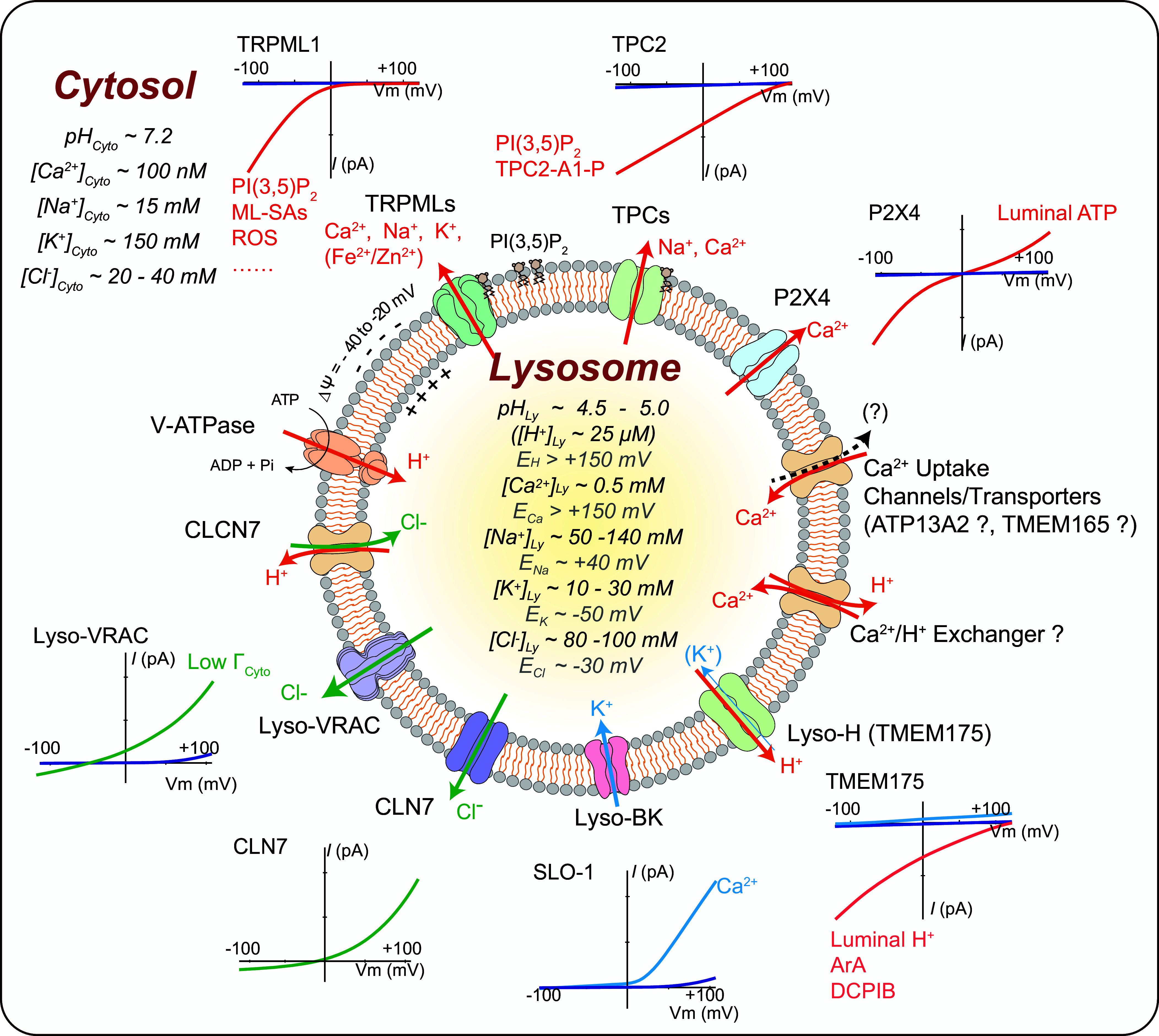
Ion channels in the lysosome. Compared with the cytoplasm, the lysosomal lumen contains high H^+^, Ca^2+^, Na^+^, and Cl^−^ but low K^+^. Lysosomal membrane potential (Δψ = ψ_Cytosol_ − ψ_Lumen_) is cytosolic side negative, with Δψ ranging from −20 to −40 mV at resting conditions. Whereas the luminal acidity [pH ∼4.5–5.0, equilibrium potential of H^+^ (*E*_H_) greater than +150 mV] is established and maintained by vacuolar-type (V-)ATPase, the lysosomal Ca^2+^ gradient (>5,000-fold, *E*_Ca_ greater than +150 mV) was proposed to be established by an unidentified Ca^2+^ uptake channel/transporter, for which ATP13A2 or TMEM165 was the proposed candidate. Seven endogenous lysosomal channels have been characterized with whole late endosome and lysosome (LEL) patch clamp. Mucolipin TRP channels (TRPMLs) are Ca^2+^-permeable channels activated by phosphatidylinositol 3,5-bisphosphate [PI(3,5)P_2_], TRPML-specific synthetic agonists (ML-SAs), and reactive oxygen species (ROS); two-pore channels (TPCs) are highly Na^+^-selective channels when activated by PI(3,5)P_2_/TPC2-A1-P, but the channels’ weak Ca^2+^ permeability may increase significantly when activated by NAADP/TPC2-A1-N; P2X4 is a luminal ATP-activated nonselective cation channel in the lysosomes of some cell types; Lyso-big-conductance calcium-activated K^+^ (BK)/LysoK_VCa_ is a lysosomal Ca^2+^-activated voltage-dependent K^+^ channel; Lyso-volume-regulated anion channel (VRAC)/LRRC8A is a low osmolarity-activated Cl^−^ channel; TMEM175 is a proton-activated proton-selective channel (LyPAP) with slight K^+^ permeability under the condition of acidic lysosomes; CLN7 was recently reported to function as a lysosomal Cl^−^ channel. Representative current (*I*)-membrane potential (*V*_m_) curve of each lysosomal channel is shown alongside the cartoon illustration of the channel. ArA, arachidonic acid.

**FIGURE 4.**
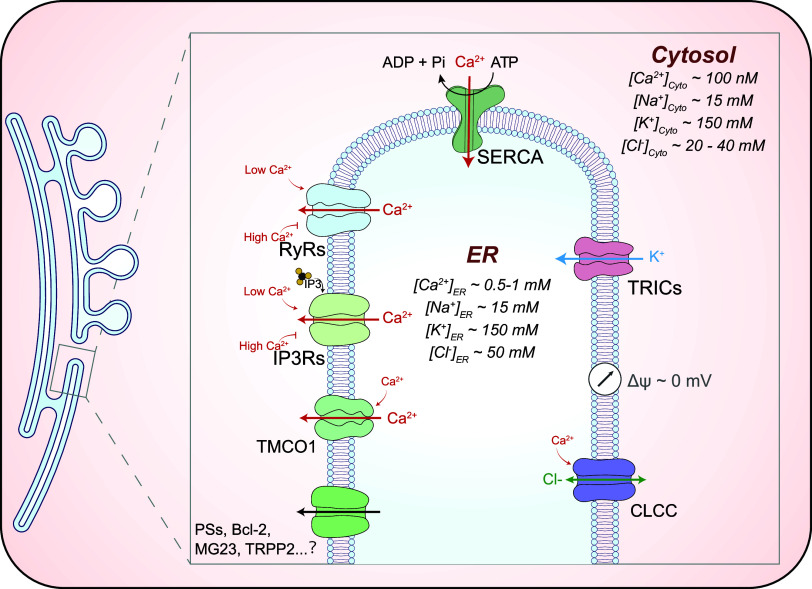
Ion channels in the endoplasmic reticulum (ER). The ER is the largest intracellular Ca^2+^ store, with a luminal concentration of 0.5–1 mM. The Ca^2+^ gradient is established by the sarco(endo)plasmic calcium ATPase (SERCA) pump. Given that membrane potential (Δψ) is ∼0 mV, equilibrium potential of Ca^2+^ (*E*_Ca_, greater than +150 mV) is the primary driving force for Ca^2+^ efflux/release. ER Ca^2+^ release is mainly mediated by 2 families of Ca^2+^-permeable ion channels: inositol (1,4,5)-trisphosphate (IP_3_) receptors (IP3Rs) and ryanodine receptors (RyRs). The activity of IP3Rs and RyRs is regulated by a variety of ligands, most notably Ca^2+^ and IP_3_. Cytosolic Ca^2+^ regulates the activity of IP3Rs and RyRs in a biphasic manner: a low concentration of Ca^2+^ ([Ca^2+^]_Cyto_) is stimulatory, but a high concentration is inhibitory. Several ER membrane proteins, including Presenilins (PSs), Bcl-2, Mitsugumin 23 (MG23), and TRPP2, have been postulated to serve as additional Ca^2+^ “leak” channels; TMCO1 is an ER Ca^2+^ channel activated by high [Ca^2+^]_ER_. In addition, whereas trimeric intracellular cation channels (TRICs) are ER K^+^-permeable channels, CLCC1 is an ER Cl^−^ channel. Both TRICs and CLCC1 were believed to conduct counterion currents to facilitate ER Ca^2+^ release.

**FIGURE 5.**
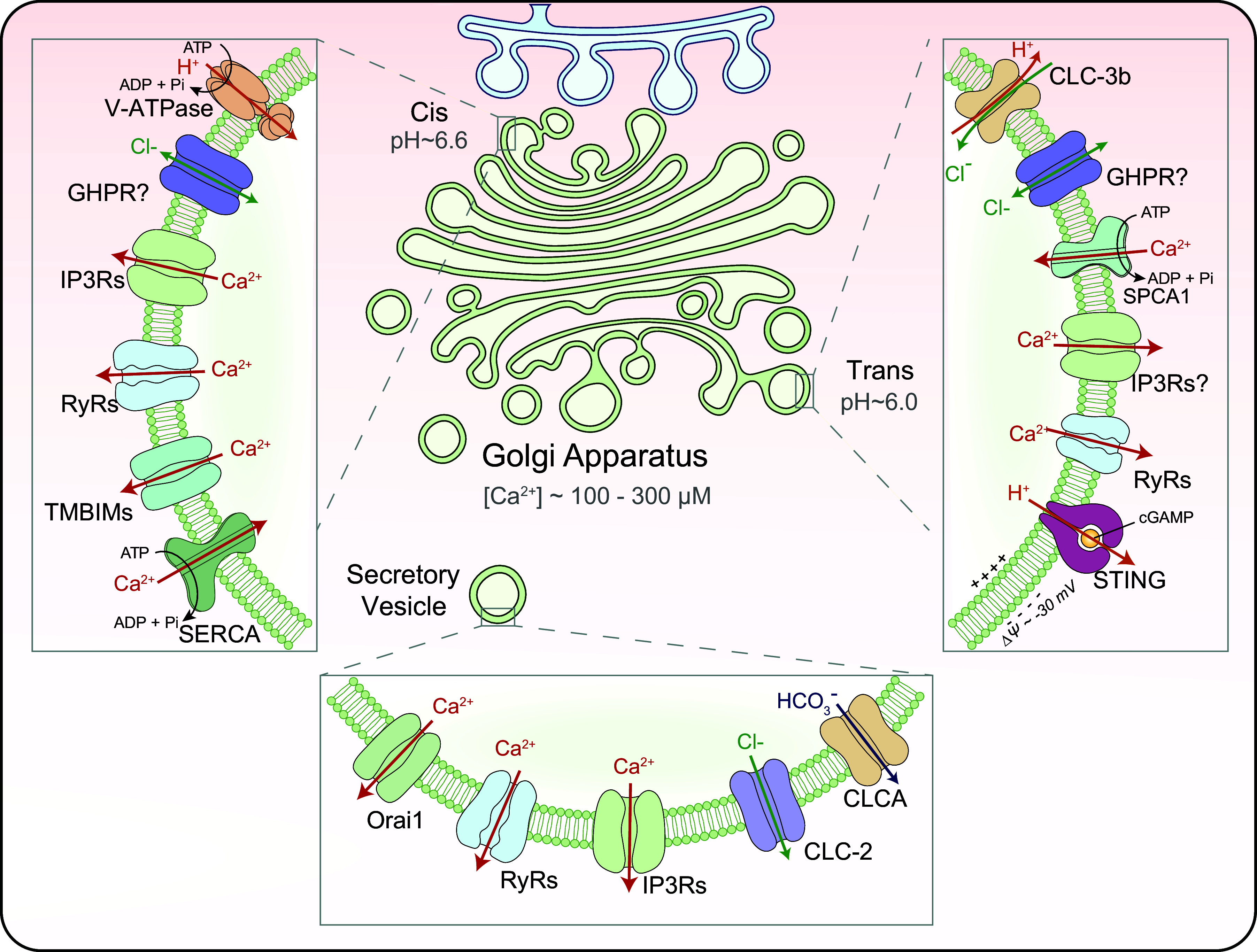
Ion channels in the Golgi apparatus and secretory vesicles. The Golgi apparatus is a high-Ca^2+^ and low-pH compartment. The transmembrane Ca^2+^ gradient is established and maintained by two Ca^2+^ pumps: sarco(endo)plasmic Ca^2+^ ATPases (SERCAs) and secretory pathway Ca^2+^ ATPases (SPCAs). The Ca^2+^ release pathways include inositol (1,4,5)-trisphosphate (IP_3_) receptors (IP3Rs) and ryanodine receptors (RyRs), and possibly transmembrane BAX inhibitor motif proteins (TMBIMs). The luminal acidity (pH 6.0–6.6) is established and maintained by vacuolar-type (V-)ATPase. STING is proposed to be a H^+^ channel/transporter in the Golgi. ClC-3b (Golgi-localized ClC-3 variant) and GPHR (Golgi pH regulator) proteins were postulated to provide Cl^−^ as the counterion for continuous electrogenic V-ATPase pumping. In secretory vesicles, several Ca^2+^-permeable channels, which include RyRs, IP3Rs, and Orai1 and two anion channels, CLCA and CLC-2, are reportedly present.

In this review, we aim to summarize the recent developments in our understanding of organellar channels in the endomembrane, with a focus on the ion channels in the ER and lysosomes, which have been most extensively studied (36, 37, 56, 57). The rapid progress made in this area of research has benefited largely from organelle-targeted electrophysiological methods and fluorescence-based imaging assays (FIGURE 2), leading to functional identification of new organellar channels (FIGURE 3, FIGURE 4, AND FIGURE 5) (2, 24, 33, 34, 41). Additionally, improved proteomic and genomic approaches have also contributed to the discovery of new organellar channels (2, 58–60). Furthermore, with the knowledge that organellar channel dysfunction causes various diseases, advances in the understanding of organellar channels have led to the identification of novel targets for therapeutics. For example, lysosomal channels have now become “druggable targets” for lysosome storage diseases (LSDs) and common neurodegenerative diseases such as Parkinson’s disease (PD) and Alzheimer’s disease (AD) (13, 57, 61–64). Together, these recent improvements have provided an updated “toolkit” for studying endomembrane channels (24, 32, 65, 66) as well as ion channels in other intracellular membranes, e.g., mitochondria, which are reviewed elsewhere (see Ref. 67).

### 1.2. Ion Gradients across Organellar Membranes

Luminal ionic composition varies greatly in different endomembrane organelles. For example, [Ca^2+^]_Lumen_ is high in the ER, Golgi apparatus, and lysosomes but relatively low in early endosomes and nascent autophagosomes (4, 24, 68) (FIGURE 1). Nevertheless, with the exception of the nucleus, all endomembrane organelles are considered intracellular Ca^2+^ stores, with the ER and lysosomes especially important (4, 24, 26) (FIGURE 1). Likewise, [K^+^]_Lumen_ is high in most secretory organelles such as the ER, the nucleus, and the Golgi apparatus (>100 mM) but low in the degradative organelles such as phagosomes, late endosomes, and lysosomes (<30 mM; see FIGURE 3) (2, 13, 25, 69–71). Hence, with [K^+^]_Cytosol_ of ∼150 mM, the equilibrium (Nernst) potential of K^+^ (*E*_K_) across organellar membranes is either close to 0 mV or between –80 and –40 mV (we use a value of approximately –50 mV for the sake of discussion in the present review) depending on the organelles. In contrast, [Na^+^]_Lumen_ is low in the cytosol and nascent autophagosomes (10–20 mM) as well as in the nucleus, the ER, and the Golgi apparatus (<20 mM) but high in the LELs (50–140 mM; we use a value of ∼100 mM for the sake of discussion; see FIGURE 3) (36, 69). With [Na^+^]_Cytosol_ of ∼15 mM, *E*_Na_ is either close to 0 mV or approximately +40 mV (value assigned for the sake of discussion) depending on the organelles (2, 25, 36). [Cl^−^]_Lumen_ is high in the lysosomes (80–100 mM) but low in other endomembrane organelles (40–60 mM) (52, 72). Hence, *E*_Cl_ is approximately –25 mV in the lysosomes and less for endosomes. [H^+^]_Lumen_ is high for all the so-called acidic compartments, e.g., the Golgi apparatus (pH 6.0–6.6), secretory vesicles (pH ∼5.7), endosomes (pH 5.5–6.3), and lysosomes (pH 4.5–5.0, *E*_H_ greater than +150 mV) but low (close to the neutral pH of 7.2) in other organelles (FIGURE 1) (28). As the fluid contents of nascent autophagosomes are derived from the cytosol, the equilibrium potential of the ion (*E_x_*, *x* = specific ion) is likely to be 0 mV for most ions (25). However, the existence of Ca^2+^ channels in autophagosomes suggests that ion gradients, at least for Ca^2+^, are likely to be gradually established during autophagosome maturation (73). Likewise, luminal ionic compositions may differ significantly for phagosomes in the early versus late stages of maturation (74, 75).

Importantly, in small-sized organelles like lysosomes, the luminal concentration of one ion must be viewed in the context of the flux of other ions. Because of the presence of various ion cotransporters in organelles, an increase in the permeability of one ion may alter the concentration gradients of others (13, 25). Hence, unlike their plasma membrane counterparts, the opening of one organellar ion channel may have a direct and transient influence on organellar Δψ and the luminal concentration of the ion, indirectly affecting the functions of other organellar channels (13, 25, 71, 76). Additionally, membrane fusion events, e.g., the fusion of high-K^+^ but low-Na^+^ organelles, e.g., autophagosomes, with those containing high Na^+^ but low K^+^, e.g., lysosomes (51), may rapidly change the ionic composition of the resulting organelle, such as autolysosomes and endolysosomes. Furthermore, the luminal ionic compositions for individual small-sized vesicles may vary dramatically in cells, depending on the recent activities of organellar channels and transporters in the same vesicles (13, 71, 76). Hence, it is expected that there is a broad distribution in the luminal concentrations of ions in the cell. For instance, lysosomal [Na^+^] varies significantly with the activities of lysosomal Na^+^ channels (76). Therefore, although we use the average values in this review if the measurements have not been made definitively, we need to realize that up to severalfold variations are possible for most organellar ions. As luminal ionic compositions are determined either in situ in the cells or in vitro on isolated organelles, often from different studies that used different cell types, for the sake of discussion we use judicious estimates of the average values throughout the present review.

#### 1.2.1. Ca^2+^.

Ca^2+^ efflux from ER, endosomes, and lysosomes is thought to be important for signal transduction, organelle homeostasis, and organelle trafficking (7, 42, 69, 77–79). In the ER, [Ca^2+^]_Lumen_ is thought to be 0.5–1 mM (ER *E*_Ca_ greater than +150 mV), which is established by the sarco(endo)plasmic reticulum calcium ATPase (SERCA) (4). Additional Ca^2+^ transporters such as secretory pathway Ca^2+^ ATPase 1 (SPCA1) may help establish the Ca^2+^ gradients across the Golgi membranes (27, 80). In the lysosomes, [Ca^2+^]_Lumen_ is ∼0.5 mM, ∼5,000-fold higher than [Ca^2+^]_Cytosol_ (∼100 nM; lysosomal *E*_Ca_ greater than +150 mV) (24, 68, 81, 82). The lysosomal Ca^2+^ gradient is established by an unknown mechanism, which might use putative Ca^2+^ transporters like TMEM165 (83, 84) or ATP13A2 (68, 69). Although both proteins may transport additional substrates other than Ca^2+^, [Ca^2+^]_Lysosome_ was found to be dramatically reduced in cells lacking TMEM165 or ATP13A2 (68, 83, 84). Because ER Ca^2+^ may provide the source of Ca^2+^ that can be taken up by lysosomes at membrane contact sites (MCSs), the putative Ca^2+^ uptake transporter can be a low-affinity one (26, 85). However, measurement of Ca^2+^ uptake and release directly with in vitro preparations, e.g., isolated lysosomes, is still lacking.

Cellular functions that are attributed to organellar Ca^2+^ release are defined with the so-called BAPTA versus EGTA test (7, 78). Although both BAPTA and EGTA have high binding affinities for Ca^2+^, BAPTA binds to Ca^2+^ ∼100 times faster than EGTA does (77). Therefore, a distinct sensitivity to BAPTA versus EGTA suggests that the source of Ca^2+^ must be spatially close to the action spot (7, 42, 77, 86). In other words, organelles themselves most likely provide the Ca^2+^ required for the specific cellular function (25, 77, 87). For instance, in cell-free vesicle fusion assays, late endosome-lysosome fusion is inhibited by BAPTA but not EGTA (7, 87). Likewise, preloading cells with BAPTA-AM (the ester form of BAPTA that is membrane permeant), but not EGTA-AM, blocks lysosomal exocytosis (88), retrograde transport of lysosomes (89), and ER-dependent autophagosome formation (86). The existence of multiple Ca^2+^ sensors/effectors may allow lysosomal Ca^2+^ release to regulate distinct steps of lysosomal trafficking and ER/sarcoplasmic reticulum (SR) Ca^2+^ release to regulate various functions, ranging from muscle contraction to autophagosome biogenesis (3, 86). For lysosomal exocytosis, the C2-domain-containing Synaptotagmin-VII (Syt-VII) is likely the Ca^2+^ effector (88, 90). For lysosomal membrane fusion and fission, the candidate effectors include calmodulin (CaM) (87, 91) and ALG-2, a lysosome-targeted EF-hand protein (89, 92, 93). The ER Ca^2+^ sensors include extended Synaptotagmin family proteins (E-Syts) for membrane contact, troponin C for muscle contraction, and calcineurin for transcription factor NFAT (3–5).

#### 1.2.2. Na^+^/K^+^.

Because Na^+^ and K^+^ are the major cations in cell physiology that display an inverse relationship in their abundance in many cellular compartments, they are discussed together. For the ER and the Golgi apparatus, [Na^+^]_Lumen_ and [K^+^]_Lumen_ are posited to be similar to [Na^+^]_Cytosol_ and [K^+^]_Cytosol_ (94–97); hence *E*_Na_ and *E*_K_ are close to 0 mV for these organelles (see FIGURE 4). For endosomes and lysosomes, although earlier studies using indirect measurements (e.g., null-point titration) suggested that the lysosomal lumen is a high-K^+^ compartment (hence little to no Na^+^/K^+^ concentration gradient) (69, 98), large lysosomal concentration gradients for both Na^+^ and K^+^ were reported based on inductively coupled plasma mass spectrometry (ICP-MS) measurement of isolated lysosomes (13, 51). Although still controversial, given the existence of multiple Na^+^- and K^+^-selective channels now discovered in the lysosomes, the balance of opinion is that a high-Na^+^ lumen is more likely to reflect the scenario in the native lysosomes, i.e., both *E*_Na_ and *E*_K_ are significant (13, 36). Hence, endolysosomal Na^+^ and K^+^ conductance may help set the membrane potential (Δψ) of endosomes and lysosomes (13, 45, 46, 99). In addition, for acidic organelles organellar Na^+^ and K^+^ flux are also critical determinants of the organellar acidification rate (100). For the lysosomal lumen, the reported and estimated values range from 50 to 140 mM for Na^+^ and from 10 to 30 mM for K^+^ (13, 36, 51, 69, 71, 76, 98). A very recent organellar ion imaging study revealed that lysosomal [Na^+^] is highly heterogeneous for individual lysosomes, with an average value between 50 and 70 mM (76). Hence, lysosomal *E*_Na_ and *E*_K_ are posited to be about +40 mV and −50 mV, respectively (FIGURE 3). The mechanisms by which Na^+^/K^+^ gradients are established or maintained are largely unknown. Although endocytosis and phagocytosis may provide Na^+^ to the lumen, endosomal H^+^-coupled Na^+^/K^+^ exchangers (NHEs) may regulate the luminal Na^+^ concentrations in the early and late endosomes and possibly lysosomes (36, 69, 76, 101).

#### 1.2.3. H^+^.

Both the ER and the nucleus have a neutral luminal pH (pH_Lumen_ ∼7.2), the same as the cytosolic pH of 7.2 (28). In contrast, the luminal acidities of endosomes and lysosomes (pH_Lumen_ ∼4.6 for lysosomes, pH_Lumen_ ∼5.5 for late endosomes, pH_Lumen_ ∼6.3–6.5 for early and recycling endosomes), as well as for the Golgi apparatus (pH_Lumen_ ∼6.0–6.6) and secretory vesicles (pH_Lumen_ ∼5.7), are established and maintained mainly by the vacuolar-type ATPase (V-ATPase) but also by some secondary H^+^ transporters (28, 29, 94, 100) (FIGURE 1). In many cells, the basal activities of Cl^−^/Na^+^/K^+^/Ca^2+^ channels/transporters are stimulated by ambient levels of cellular cues and may contribute to acidification by providing counterion flux for continuous electrogenic V-ATPase pumping (13, 29, 36, 72, 100, 102). On the other hand, there are also multiple H^+^ efflux pathways, which include NHEs for early and late endosomes (101), transmembrane protein 175 (TMEM175) H^+^ channels, and unidentified H^+^-coupled transporters for lysosomes (28, 103–105). For lysosomes, in addition to assisting hydrolases, the established 500- to 1,000-fold H^+^ gradient is also important for Δψ generation, lysosomal trafficking, and content condensation during membrane fission (7, 69, 75, 106). The lysosomal H^+^ gradient also provides the driving force for many H^+^-dependent catabolite exporters (36, 107). Finally, the V-ATPase is also essential for the lysosome to sense the luminal amino acid levels (17). Although juxtaorganellar H^+^ release plays a role in the regulation of organellar functions, e.g., lysosomal trafficking, the H^+^ sensors/effectors in these processes have not been firmly established in most cases (28, 30).

#### 1.2.4. Cl^−^.

[Cl^−^]_Lumen_ is estimated to be 80–100 mM in lysosomes, 60–80 mM in late endosomes, and 40–50 mM in early endosomes, the ER, and the Golgi apparatus, compared with 110 mM in the extracellular space and 20–40 mM in the cytosol (52, 53, 72, 108, 109). Therefore, *E*_Cl_ is −15 mV for the early endosomes, the ER, and the Golgi apparatus and approximately −30 mV for lysosomes. Whereas lysosomal Cl^−^ flux regulates lysosomal Δψ and endolysosomal Ca^2+^ release (52, 110), high [Cl^−^]_Lysosome_ is required for lysosomal hydrolase activation, with some hydrolases acting as Cl^−^ sensors (52–55). The high intralysosomal Cl^−^ is established by CLCN7/ClC7, a H^+^/Cl^−^ exchanger that functions as a H^+^-dependent Cl^−^ transporter in the lysosome (53, 55, 111).

#### 1.2.5. Fe^2+^/Zn^2+^.

In addition to the abundant ions, there are also trace metal ions present in the lumens of ER and lysosomes. For instance, lysosomes are the intracellular stores of Fe^3+^/Fe^2+^/Zn^2+^/Cu^2+^ in micromolar concentrations, and there exist various Fe^2+^/Zn^2+^ ion channels and transporters in the lysosomes (33, 112–114).

#### 1.2.6. Membrane potential.

Membrane permeabilities of ions down the concentration gradients determine the membrane potential (Δψ) of the cell as well as that of the endomembrane organelles. At the plasma membrane, Δψ is defined as ψ_Cytosol/Inside_ − ψ_Extracellular/Outside_, where ψ_Extracellular_ is set to 0 mV in the electrophysiological experiments in which the bath solution is connected to the ground wire, such that the resting Δψ is around −70 mV (−70 mV to 0 mV) for most cells. In organellar patch-clamp experiments (see sect. 2), Δψ could be defined as ψ_Lumen/Inside_ − ψ_Cytosol/Outside_ if an organelle is treated as a “cell,” where ψ_Cytosol/Outside_ is set to 0 mV as the bath/cytosolic solution is connected to the ground wire. However, in the cells, given that the extracellular side is equivalent to the luminal side in both secretory and endocytic vesicles (13), endomembrane Δψ is defined as ψ_Cytosol_ − ψ_Lumen_ in the present review, so the knowledge gained from whole cell studies can be extrapolated for endomembrane channels that are also dually localized at the plasma membrane (45, 46, 115). Theoretically, cation influx into the organellar lumen and anion efflux from the lumen may contribute to a negative organellar Δψ, whereas cation efflux from the lumen and anion influx into the lumen may contribute to a positive organellar Δψ. Any ions with their *E_x_* values different from the “resting” organellar Δψ would cause changes to organellar Δψ if the specific channel opens. “Resting” Δψ is posited to be ∼0 mV for the ER and the nucleus and slightly negative (−20 to −40 mV, −30 mV for the sake of discussion) for the Golgi apparatus, phagosomes, and lysosomes, although more negative Δψs have also been reported for lysosomes and the Golgi apparatus (13, 106, 116, 117). Whereas isolated lysosomes have resting Δψ ∼0 mV under the presumed ionic compositions in the absence of cellular cues, in intact cells the V-ATPase may help establish a luminal side positive Δψ (i.e., negative Δψ) (45, 106). In the native lysosomes of the cell, the modest Δψ of approximately −30 mV is maintained by various “background” ionic membrane permeabilities in the cell (13, 53), as more negative lysosomal Δψ (e.g., when the V-ATPase activity is unopposed) would adjust their contributions to Δψ and cause an inhibition of the V-ATPase activity, both in a negative-feedback manner (29, 53, 100). Note that the basal activities of the background channels could be induced and maintained by the ambient levels of cellular cues that activate these channels (13). As lysosomal *E*_Ca_ (greater than +150 mV) and *E*_Na_ (approximately +40 mV) are more positive than resting lysosomal Δψ, yet lysosomal *E*_K_ (approximately −50 mV) is more negative than resting lysosomal Δψ, the opening of lysosomal Na^+^ and Ca^2+^ channels would depolarize or even reverse the lysosomal Δψ, whereas opening of lysosomal K^+^ channels would hyperpolarize the lysosomal Δψ. As lysosomal *E*_Cl_ is close to resting lysosomal Δψ, lysosomal Cl^−^ channels may play a role in Δψ stabilization (see FIGURE 3). On the other hand, the driving force of ion flux is determined by both the organellar Δψ and the ion gradient across the organellar membrane, i.e., the electrochemical gradient. Hence, a change of organellar Δψ, e.g., when the inhibition of V-ATPase results in a depolarization of lysosome Δψ (106), may decrease the driving force for TRPML1-mediated lysosomal Ca^2+^ release (62, 85).

### 1.3. Ion Channels in the Endomembrane

Ion channels and transporters in the endomembrane organelles provide the ionic environment necessary for protein folding, degradation, pathways for import and export, and signals for vesicular trafficking (2, 13, 22, 39). The existence of large ion gradients suggests that channels and transporters exist on the endomembranes. Tens of ion channels are reportedly present in endomembrane organelles based on molecular expression analysis, pharmacological and genetic manipulation, or functional characterization (2, 36, 39). In the present review, we mostly focus on those supported by strong data from all three approaches. For molecular expression analysis, it is important to note that whereas some channel proteins are present in the organelles as resident proteins, others might just be transmembrane proteins in intraluminal vesicles undergoing degradation (78). Therefore, it is important to show that protein expression is on the limiting membranes of degradative organelles, which has been made possible with the advance of superresolution microscopy. Alternatively, endosomes and lysosomes can be enlarged by various genetic and pharmacological means (32, 118) so that localization on the perimeter membranes can be convincingly demonstrated. Because cargo membrane proteins undergo constant membrane trafficking, for the resident organellar channels the “dwell time” on the limiting membrane must be high for the enrichment of expression to be observed.

Whereas some organellar channels are targeted specifically and exclusively to one organelle, i.e., it has a very high level of enrichment (we refer to them as committed organellar channels), others are present in both plasma membrane and intracellular compartments [we refer to them as noncommitted organellar channels (13, 56)]. Hence, for the noncommitted organellar channels, it is necessary to set up the criteria to define the functions of organellar versus plasma membrane channels. It is also important to note that in an overexpression experiment committed organellar channels may also appear in nonresidential locations and thus take on nonphysiological roles (13). Hence, it is important to study the organellar channels in their native settings at native expression levels. In this review, we focus on endomembrane channels that are functionally studied with organelle physiology, especially in the endogenous expression setting, but when the data are absent we briefly discuss the current state of knowledge but call for organelle electrophysiology studies. For the channels that have already been electrophysiologically studied in nonnative settings, we compare these results with complementary assays, e.g., fluorescence-based ion imaging in intact cells. In this regard, since IP3Rs and ryanodine receptors (RyRs) are highly enriched in the ER, they are considered to be bona fide ER channels whose ER functions have been clearly established (37). Nevertheless, direct organellar patch-clamp studies on them are still desired. In contrast, some plasma membrane channel proteins may appear on the ER membranes, but this is during the early steps of their biogenesis en route to their plasma membrane destination (39). Such channels are unlikely to be active in the ER even though their ER localization can be shown by molecular expression analyses. Likewise, endogenous TRPML1 and TMEM175 channels are almost exclusively enriched on the limiting membranes of LELs and are hence bona fide lysosomal channels, with their lysosomal functions being clearly established (36, 44, 104). However, some plasma membrane channel proteins may be detected in the lysosomes but as a degradation cargo in the lumen (e.g., in the intraluminal vesicles), meaning they are not on the limiting membranes of lysosomes to a significant degree. Such channel proteins are unlikely lysosome-resident membrane proteins active in the lysosomes, and they should not be considered “lysosomal” channels.

Although some organellar channels may be constitutively active, most of them are activated by specific cellular cues (13, 51, 104). When low-level channel activity is present in native cells, it is often unclear whether this is because the channel is a “leak” channel (with some activity in the absence of any stimulus) or it is because there is a low (ambient) level of the endogenous activator present under basal conditions. For example, IP3Rs may be stimulated by basal activation of PLC, which produces an ambient level of IP_3_ (119). IP3Rs may thus appear to function as Ca^2+^ leak channels. In the lysosomes, there may exist leaklike Na^+^/K^+^ (99) and H^+^ (103, 104) conductance. However, the H^+^ leak channel is activated by the endogenous level of luminal protons (103, 104), and the lysosomal Na^+^/K^+^ channels are regulated by the activity of mTOR, whose activity depends on the metabolic status of the cells (15). It makes sense that lysosomal channels are not constitutively active, because H^+^ and Ca^2+^ are 1,000–5,000 times more abundant in the lysosome lumen than in the cytosol, meaning there is a large *E*_H_ and *E*_Ca_ across the lysosomal membrane; therefore, the pathways for H^+^ and Ca^2+^ flux, i.e., Ca^2+^- and H^+^-permeable channels, must be tightly regulated (13, 24, 104). The cellular cues that activate endomembrane organellar channels can be very diverse, including Ca^2+^, H^+^, lipids, nucleotides, interacting protein partners, and kinases (13, 37, 40). Whereas some activating cues act on the cytosolic side, others act on the luminal side or within the organellar membrane (45, 104, 120, 121).

Several major ionic conductances have now been recorded in their native membranes with organellar electrophysiology (2, 34, 36, 122) (FIGURE 2*A*). In the present review, we focus on the organellar conductances whose molecular identities are known, such that their physiological functions can be revealed with both in vitro and in vivo assays. Significantly, molecular and genetic studies have in turn provided definitive evidence for the existence of these conductances, confirming their previously proposed cellular and biological functions. Decades of organelle channel research have now revealed the existence of cation-selective and -nonselective channels for Ca^2+^, Na^+^, K^+^, Zn^2+^, Fe^2+^, and H^+^, as well as anion channels for Cl^−^ on endomembranes (FIGURE 3, FIGURE 4, AND FIGURE 5) (2, 33, 37, 45, 46, 49, 51, 57, 110, 115, 123). In this review, we discuss all endomembrane channels, but with emphasis on Ca^2+^-permeable channels and channels in the well-studied ER and lysosomes.

## 2. METHODS TO STUDY ENDOMEMBRANE CHANNELS

There are common challenges in studying channels from different intracellular organelles. Unlike plasmalemmal channels, whose working environment has been unambiguously defined, the basic information for most organelles has yet to be definitively established, which includes luminal ionic composition, organellar membrane potential, and lipid composition of the organellar membranes.

### 2.1. Organellar Patch-Clamp Electrophysiology

There are two types of organellar channels: committed versus noncommitted organellar channels. For the latter, because those channels can traffic between the plasma membrane and intracellular organelles, it is possible to characterize their channel properties with whole cell recordings (78) and then make extrapolations to the organellar membranes. However, for the committed organellar channels, even if the expression at the plasma membrane can be induced in an overexpression system, because the basic properties of organellar membranes may differ significantly from the plasma membrane, it is necessary to characterize the functions of intracellular channels in their native environment (32–34, 51, 65, 124). One of the biggest hurdles to characterizing intracellular channels in their native membranes is the relatively small size of intracellular organelles/vesicles. For example, endosomes and lysosomes are usually <0.5 µm in diameter, which is suboptimal for patch-clamping studies. However, endolysosomes can be enlarged by genetic and pharmacological means (32, 33, 51, 65, 124). For example, early endosomes can be enlarged by transfecting the cells with constructs that are known to affect endosomal membrane trafficking, e.g., using a mutant form of the AAA ATPase SKD1/VPS4 (124) or a constitutively active mutant form of Rab5 (32, 125) (see FIGURE 2*A*). Such maneuvers increase the size of early endosomes to a patchable range (3–6 μm in diameter); the cell membrane can be sliced open manually by using electrodes to release enlarged endosomes (32, 34) (see FIGURE 2*A*). On the other hand, LELs can be easily enlarged by chemical approaches (33). For example, vacuolin-1 is a small molecule that can induce the formation of enlarged LELs via unclear mechanisms (118). After exposure to vacuolin-1 for several hours, large LELs (up to 3–6 μm in diameter) can be isolated for whole LEL recordings (33). Overall, endosomes and lysosomes may be enlarged to a patchable range via genetic, pharmacological, and physiological means (see FIGURE 2*A*). Four distinct configurations can be achieved for LEL recordings: LEL attached, luminal side out, cytosolic side out, and whole LEL (32–34, 51, 65, 124). In the whole LEL configuration, the extracellular solution in the patch pipette (electrode) can be adjusted to pH 4.6 to mimic the acidic condition of LEL (33) (see FIGURE 2*A*). Inward current is defined as cations flowing out of the LEL lumen (FIGURE 2*A*). In addition to whole LEL, whole early endosome, whole recycling endosome, and whole phagosome recordings have also been developed (32–34, 51, 65, 124). Although whole Golgi apparatus and whole ER recordings have not been developed, nucleus-attached recordings have made it possible to study nuclear IP3Rs and RyRs (66).

### 2.2. Lipid Bilayer Reconstitution and Giant Unilamellar Vesicles

An alternative way to study organellar channels is to reconstitute the channel proteins into a planar lipid bilayer (126–128) or to reconstitute the organellar membranes into giant unilamellar vesicles (GUVs) (129). The bilayer technique is commonly used to study the electrophysiological properties of a single ion channel in a defined and artificial lipid bilayer (128), although if the protein amounts are high macroscopic currents can also be detected (108). ER channels are extensively and almost exclusively studied with this approach (126–128). However, because the channels are not under their native environments, it is necessary to verify the regulatory mechanisms revealed by the bilayer studies with the use of yet-to-be-developed whole ER patch-clamp recordings. An intrinsic problem with single-channel lipid bilayer studies is the imperfect purity of the protein preparation, and hence possible contaminations by other channels (128, 129). Several lysosomal channels have been studied with the lipid bilayer or GUV approach, yet the revealed channel properties were often not able to be confirmed in whole LEL studies (25, 36, 57, 78, 129, 130). It is also important to note that bilayer recording is a sufficient assay in a nonnative environment. Hence, all the conclusions should be verified by necessity tests in the cell-based assays.

### 2.3. Organellar Ion Imaging: Fluorescent Dyes

Organellar ion flux can be fluorescently monitored in two ways: luminal versus cytosolic ion imaging (24). Ions can be imaged with ion-sensitive fluorophores and genetically encoded protein-based ion indicators. Taking Ca^2+^ as an example, two different types of Ca^2+^ indicators are commonly used to detect intracellular Ca^2+^ levels: Ca^2+^-sensitive fluorescent dyes and genetically encoded protein-based Ca^2+^ sensors (24, 41, 131, 132). For the dye approach, luminal Ca^2+^ dyes are more useful, as cytosolic Ca^2+^ dyes are generally difficult to target to the cytoplasmic side of the specific organelles. Note that [Ca^2+^] differs dramatically in the cytosol versus the lumen, with up to 5,000-fold differences. Hence dyes with different Ca^2+^-binding affinity (*K*_d_) and dynamic range should be employed; whereas cytosolic Ca^2+^ dyes should have high affinity in the tens to hundreds of nanomolar range, luminal dyes should have low affinity in the hundreds of micromolar range. Because noncommitted organellar Ca^2+^ channels are present in both the plasma membrane and intracellular membranes, to exclude the possibility of extracellular Ca^2+^ entry cytosolic Ca^2+^ imaging experiments should be performed in a Ca^2+^-free bath solution (85, 133). More importantly, the organellar Ca^2+^ source can be confirmed if the Ca^2+^ uptake mechanism in the organelles is already known, e.g., using SERCA pump inhibitors to deplete ER Ca^2+^ stores (37, 85, 86). Likewise, the LEL source of Ca^2+^ can be confirmed by using glycyl-l-phenylalanine 2-naphthylamide (GPN) to deplete or partially deplete the lysosomal Ca^2+^ stores (26, 85, 134).

Luminal ion dyes are more useful for studying organellar channels because it is possible to target the dyes to the organelles, e.g., through endocytosis (24, 41). In addition, DNA-conjugated Ca^2+^ fluorophores have also been developed to study organellar Ca^2+^, in which specific organelle targeting can be achieved, especially with the endocytic organelles (68). By using various ionophores in combination to permeabilize the cells, such that the concentration of a luminal ion is based on its concentration in the medium or bath solution (135–137), it is also possible to calibrate the luminal concentrations of ions. For example, [Ca^2+^]_Lysosome_ ranges from ∼0.35 mM by single-lysosome measurement to ∼0.5 mM by cell-averaged measurement (68, 81). Upon activation of lysosomal Ca^2+^ channels, a decrease in [Ca^2+^]_Lumen_ can be observed with dextran-loaded luminal Ca^2+^ dyes with high micromolar *K*_d_ (24, 81, 82). Similarly, lysosomal pH is studied with dextran-conjugated pH-sensitive dyes (e.g., p*K*_a_ ∼4.7 for Oregon Green 488) (28, 98), and [Cl^−^]_Lumen_ can be measured and calibrated with dextran-loaded and DNA targeting-based Cl^−^-sensitive dyes with α *K*_d_ > 50 mM (54, 55, 108). Complementary to the dye approach, organelles purified with the density-gradient ultracentrifugation or immunoisolation approach can be used to determine the luminal ionic compositions with inductively coupled plasma mass spectrometry (ICP-MS) (51), although a caveat is that the lengthy procedures performed in vitro might affect the accuracy of the determination.

### 2.4. Organellar Ion Imaging: Genetically Encoded Indicators

Organelle-targeted genetically encoded Ca^2+^ indicators (GECIs) are a new generation of probes for studying organellar Ca^2+^ release (24, 41, 85, 131) (FIGURE 2*B*). Both luminescent and fluorescent reporter proteins change their spectral properties upon Ca^2+^ binding (24, 138). On the basis of their structure, at least three different types of GECIs exist. Whereas aequorins are naturally bioluminescent reporters, GCaMPs have a Ca^2+^-binding motif from calmodulin (CaM) that has been fused with a fluorescent reporter protein, e.g., green fluorescent protein (GFP), so that Ca^2+^ binding will change the fluorescence (24, 41, 139). Additionally, Cameleon proteins have a Ca^2+^-binding motif inserted between two different reporters so that Ca^2+^ binding can be measured by monitoring the efficiency of fluorescence resonance energy transfer (FRET) (140). One major advantage of GECIs over fluorescent dyes is that GECIs can be targeted to desired organelles by fusing the construct to organelle-specific targeting motifs (41, 85, 86, 132). Hence, GECIs can be used to detect Ca^2+^ levels in different endomembrane organelles, such as the ER and lysosomes (41, 62, 86). Importantly, like the dyes, whereas low-affinity (in the micromolar range) GECIs allow luminal Ca^2+^ detection if localized to the luminal side of the organellar membrane proteins, high-affinity (tens to hundreds of nanomolar range) GECIs allow juxtaorganellar Ca^2+^ measurement if localized to the cytosolic side of the organellar membrane proteins (FIGURE 2*B*). Like Ca^2+^, both luminal pH and juxtaorganellar pH can be monitored with organelle-targeted pH dyes or genetically encoded pH indicators, e.g., pHluorin and superfolder GFP (sfGFP) (104, 141) (see FIGURE 2*B*). In addition, K^+^ and Cl^−^ probes in the ER and *trans*-Golgi network (TGN) and Na^+^ probes in the endosomes and lysosomes have also been developed (70, 76, 108). In all cases, ratiometric measurement can be made possible by additionally engineering an ion-insensitive fluorescent group in the same indicator (24, 41, 108).

### 2.5. Organellar Voltage Imaging

Although electrophysiological current-clamp methods can be used to study organellar Δψ for isolated organelles, e.g., isolated lysosomes (15, 34, 45, 110), it is necessary to monitor the changes of organellar Δψ in situ in intact cells in response to cellular cues (13). Voltage-sensitive dyes have been used to measure phagosomal Δψ (116). Organelle-targeted genetically encoded voltage indicators (GEVIs) have also been developed to study Δψ in lysosomes and the Golgi apparatus (117). Recently, DNA-based voltage indicators have also been developed to study organellar Δψ (106). Such methods could be used to measure both resting Δψ as well as stimulus-induced changes in organellar Δψ (13, 106). Now that organellar channels have been molecularly identified, genetic manipulations, e.g., knockout (KO) and overexpression, can be used in combination to investigate their roles in organellar Δψ regulation. For example, expression and activation of lysosomal K^+^ channels were shown to hyperpolarize lysosomal Δψ, since lysosomal *E*_K_ is more negative than resting lysosomal Δψ (45, 49, 106). Likewise, activation of lysosomal Na^+^/Ca^2+^ channels depolarizes lysosomal Δψ, since lysosomal *E*_Na_ and *E*_Ca_ are more positive than resting lysosomal Δψ (45, 106). Furthermore, an essential role of the V-ATPase in organellar Δψ regulation has been firmly established with organellar Δψ imaging (106). Importantly, Δψ values can be calibrated both in vitro, e.g., using patch-clamped isolated organelles, and in vivo by controlling the organellar ionic compositions with a combination of ionophores (106, 117).

## 3. ION CHANNELS IN THE BIOSYNTHETIC PATHWAY

### 3.1. Ion Channels in the ER and the Nuclear Membrane

The ER, the largest membrane-bound organelle with a continuous intraluminal space, is the primary site of biosynthesis and protein folding in eukaryotic cells (6, 22). Whereas the nuclear envelope flattens around the nucleus, peripheral ER contains two distinct structural domains: ER sheets and ER tubules (5, 6) (FIGURE 4). The sarcoplasmic reticulum (SR) is a specialized form of ER in muscle cells (142). The most abundant cation in the ER/SR is K^+^, with its luminal concentration ([K^+^]_ER_) as high as [K^+^]_Cytosol_ (∼150 mM, ER *E*_K_ ∼0 mV) (96, 108) (FIGURE 4). On the other hand, [Ca^2+^]_ER_ (∼0.5–1 mM) is much higher than [Ca^2+^]_Cytosol_ (∼100 nM, ER *E*_Ca_ greater than +150 mV) (4) (see FIGURE 4). [Cl^−^]_ER_ is ∼50 mM, which is slightly higher than [Cl^−^]_Cytosol_ (10–40 mM, ER *E*_Cl_ approximately −15 mV) (2, 72, 108) (FIGURE 4). Hence Ca^2+^ is the only ER ion with an appreciable electrochemical gradient. Global ER Δψ is believed to be ∼0 mV, suggesting that the direction of ion flux is determined solely by *E_x_* (2, 37, 40). However, sizable changes of the Ca^2+^ electrochemical gradient and ER Δψ might occur transiently in local domains because of the activities of clustered ER channels and transporters (37, 122).

Because of the large volume (up to 10% of cell volume), high [Ca^2+^]_ER_, and a large amount of Ca^2+^ buffer proteins, the ER/SR is the largest intracellular Ca^2+^ store (4, 6, 79, 143). [Ca^2+^]_ER_ homeostasis is maintained by balancing Ca^2+^ uptake and efflux/release; whereas the former is mediated by SERCA, an ATP-consuming Ca^2+^ pump, the latter occurs mainly through IP3Rs and RyRs (3, 4, 37, 143) (see FIGURE 4). Electrophysiological studies of ER ion channels have mostly been conducted with lipid bilayer recordings (37, 108, 128). However, as the nuclear envelope is continuous with the peripheral ER, nucleus-attached patch-clamping has provided an alternative approach to study ER channels in a more native environment (66, 144).

#### 3.1.1. Inositol 1,4,5-trisphosphate receptors.

IP3Rs are IP_3_-gated Ca^2+^-modulated ion channels localized mainly on the ER membrane (FIGURE 4). The IP3R family contains three members, IP3R1–3, which are encoded by the ubiquitously expressed *ITPR1–3* genes (37, 145). IP3Rs are homo- or heterotetrameric channels, in which four six-transmembrane helices (S1–S6) make up the transmembrane (TM) domain that is capped by a much larger cytoplasmic domain (37, 146). IP3Rs are nonselective cation channels with a modest selectivity for Ca^2+^ over K^+^ (*P*_Ca_/*P*_K_ ∼ 6–8), yet with a large single-channel conductance (∼100 pS under physiological Ca^2+^ concentrations) (122, 147–149). Hence, localized and sizable Ca^2+^ transients may occur on the ER membranes through clustered IP3Rs (86, 122).

The channel activity of IP3Rs is regulated by a wide range of ligands, most notably Ca^2+^, IP_3_, and ATP (37, 148) (see FIGURE 4). Ca^2+^ exerts its influence on IP3Rs from both cytosolic and luminal sides (37, 148, 150). Cytosolic Ca^2+^ regulates IP3Rs, as well as the related RyRs, in a biphasic manner: whereas a low level of cytosolic Ca^2+^ stimulates the channel activity, higher concentrations are inhibitory (37, 148, 150) (see FIGURE 4). Hence, cytosolic Ca^2+^ regulates IP3Rs via a “bell-shaped” sensitivity, and there exist two cytosolic Ca^2+^ binding sites in IP3Rs with different affinities (37, 151–153). The stimulatory Ca^2+^ site consists of multiple acidic residues located in two different domains of IP3Rs, as revealed in the atomic resolution structures (37, 151, 154). The stimulatory effect of low cytosolic Ca^2+^ is suited for signal amplification via a positive-feedback mechanism, such that small local Ca^2+^ signals, e.g., from one single IP3R channel, could be amplified and propagated for signal transduction. IP_3_ itself may prime the clustering of IP3Rs, so that activation of one single IP3R channel may cause the openings of the neighboring channels (37, 153). On the other hand, the inhibitory effect of high cytosolic Ca^2+^ is believed to provide a negative-feedback regulation, thereby terminating Ca^2+^ release when the juxta-ER Ca^2+^ is sufficiently elevated to prevent depletion of the ER Ca^2+^ store (37, 146). Furthermore, the biphasic regulation of IP3Rs by Ca^2+^ may also help generate Ca^2+^ oscillations, as there are multiple Ca^2+^-dependent feedback regulatory mechanisms in place (37, 155). IP3Rs are also regulated by luminal Ca^2+^, in which high [Ca^2+^]_Lumen_ inhibits the channel activity through the luminal Ca^2+^-binding protein Annexin 1 (156).

IP_3_, produced upon PLC activation downstream of receptor stimulation, is the endogenous agonist that likely tunes the bell-shaped Ca^2+^ sensitivity of IP3Rs (40, 122, 148). In the presence of IP_3_, Ca^2+^ binding to the stimulatory site is promoted (146, 148, 153). Structural studies revealed that the IP_3_ binding site is located close to the Ca^2+^ stimulatory site, such that IP_3_ and Ca^2+^ act cooperatively as coagonists of IP3Rs (37, 151, 157). In many cells, there is a basal activity of PLC, which produces an ambient level of IP_3_ to induce a small IP3R-dependent Ca^2+^ “leak” (119). In addition, cytosolic ATP may also regulate IP3R activity (158) by binding to a site near the stimulatory Ca^2+^ site (153). In the presence of IP_3_, cytosolic ATP enhances the channel activity of IP3Rs by allosterically modulating sensitivity to Ca^2+^ (122, 158).

IP3R-mediated ER Ca^2+^ release regulates diverse cellular functions, including protein folding, secretion, gluconeogenesis, cell migration, and apoptosis, through various Ca^2+^ sensors (37, 40). IP3R-mediated Ca^2+^ oscillations may activate the Ca^2+^ sensor calcineurin to promote the nuclear translocation (hence activation) of transcription factor NFAT (3, 155). A recent study revealed that IP3R-mediated localized Ca^2+^ transients define the formation sites of autophagosomes on the ER, which provides the source membranes for phagophore expansion (86). IP3Rs may also provide Ca^2+^ to other organelles, such as mitochondria and lysosomes, through specialized membrane contact sites, thereby regulating the metabolic state of mitochondria and the refilling of lysosomal Ca^2+^ stores (4, 10, 67, 85, 159). Lysosomal Ca^2+^ release may promote the formation of ER-lysosome MCSs and activate IP3Rs on the MCSs (10, 11). Hence, IP3Rs may mediate cross talk between the biosynthetic and degradative pathways through ER-lysosome MCSs. Although the Ca^2+^ released from individual organelles is likely to act locally through signaling microdomains, upon formation of organelle-organelle membrane contact sites interorganellar Ca^2+^ signaling events may occur (10, 85, 119, 160).

#### 3.1.2. Ryanodine receptors.

The RyRs are named after the plant alkaloid ryanodine, which binds to RyRs to modulate the channel activity (161–163). In mammals, there exist three different isoforms, RyR1, RyR2, and RyR3, which are ubiquitously expressed but have high expression levels in striated muscles and neurons (161). RyRs have many similarities to IP3Rs in channel properties and regulation. Like IP3Rs, RyRs are also ER-localized 6-TM tetrameric channels with a large cytosolic cap for regulation (37). Additionally, RyRs are also ligand-gated nonselective cation channels with a large single-channel conductance (37, 161) (FIGURE 4). As IP3Rs and RyRs share similar molecular determinants for their selectivity filters, RyRs, like IP3Rs, are permeable to both monovalent and divalent cations, with a modest Ca^2+^ selectivity (37, 161).

The channel activity of RyRs is modulated by a variety of ligands, including Ca^2+^, Mg^2+^, and ATP (37). Cytosolic Ca^2+^ is the principal ligand of RyRs that affects the channel activity in a bell-shaped biphasic manner: whereas a low level of cytosolic Ca^2+^ stimulates channel activity, higher concentrations are inhibitory (37, 161). A high-affinity Ca^2+^-binding site is responsible for the stimulatory effect, as revealed in the RyR structures (164). Cytosolic Mg^2+^ exerts an inhibitory effect on RyRs, possibly by competing for the stimulatory Ca^2+^ site (37, 165). ATP enhances channel activity (37, 128); consistent with the cooperative effects of ATP and Ca^2+^, structural studies revealed that the ATP-binding site is located near the stimulatory Ca^2+^ site (164, 166).

Because RyRs are Ca^2+^-activated Ca^2+^ channels, the primary function of RyRs is signal amplification via a Ca^2+^-induced Ca^2+^ release (CICR) mechanism, which is responsible for SR Ca^2+^ release and the excitation-contraction (E-C) coupling in cardiac muscles (37). In cardiac myocytes, Ca^2+^ influx through Ca_V_1.2 could activate RyR2 to release ER Ca^2+^ (161). In contrast, in skeletal muscle, the activity of RyR1 channels is stimulated by a direct interaction between ER/SR-localized RyR1 and surface-localized Ca_V_1.1 [also known as dihydropyridine receptors (DHPRs)] (37, 167). Membrane depolarization activates RyR1 through the movement of the S4 voltage sensor of Ca_V_1.1, mediating depolarization-induced SR/ER Ca^2+^ release for E-C coupling (37, 161). In nonexcitable cells, RyRs may amplify Ca^2+^ signals from other sources, e.g., those mediated by the plasma membrane Ca^2+^ entry channels (37). Like IP3R-mediated ER Ca^2+^ release, RyR-mediated ER Ca^2+^ release also regulates diverse cellular functions including secretion through various Ca^2+^ sensors (39, 161).

#### 3.1.3. ER Ca^2+^ leak channels.

The steady-state [Ca^2+^]_ER_ is maintained at ∼0.7 mM by balancing SERCA-mediated Ca^2+^ uptake and passive Ca^2+^ efflux, i.e., Ca^2+^ leak pathways (22, 79). When the SERCA pump activity is pharmacologically inhibited, e.g., by thapsigargin, ER Ca^2+^ is quickly depleted, suggesting the existence of ER Ca^2+^ leak pathways (39, 168). Whereas RyRs, and possibly IP3Rs, can be viewed as ER Ca^2+^ leak channels, there may exist additional ER leak pathways. First, several additional ER membrane proteins are proposed to contribute to ER Ca^2+^ leak. These include Presenilins, Bcl-2, the Sec61 complex, Mitsugumin 23 (MG23), Orai3, and transient receptor potential channels (TRPV1–4, TRPM8, and TRPP2) (39, 168–174) (FIGURE 4). However, because the supporting evidence is mostly indirect or from bilayer studies, it is not clear whether these proteins regulate ER Ca^2+^ leak via IP3Rs or RyRs, or if they are bona fide ER Ca^2+^-permeable channels themselves (38, 39, 175). It is also possible that some of these ER-localized noncommitted organellar channels are regulated by specific cellular cues (39, 174).

When the ER Ca^2+^ store is overloaded, the Ca^2+^ leak pathways are expected to be facilitated, mediating the so-called store overload-induced Ca^2+^ release (SOICR) (37). Hence, there may exist a specific Ca^2+^ overload-activated Ca^2+^ conductance on the ER membrane. The activity of RyRs is stimulated by luminal Ca^2+^, possibly through a feedthrough mechanism, suggesting that RyRs may help prevent Ca^2+^ overload (37, 176). In addition, TMCO1, an ER-resident 2-TM protein, was also proposed to be a Ca^2+^ overload-activated Ca^2+^ leak channel (39, 177) (see FIGURE 4). TMCO1 was reported to exist as nonfunctional monomers in resting conditions (177). However, ER Ca^2+^ overload (i.e., high [Ca^2+^]_ER_) has been suggested to induce the formation of a tetrameric TMCO1 complex, which can function as an ER Ca^2+^ leak channel (177). Hence, in a negative-feedback manner, TMCO1 may mediate a high [Ca^2+^]_ER_-activated Ca^2+^ leak channel to prevent Ca^2+^ overload, such that resting steady-state [Ca^2+^]_Lumen_ may be the “threshold” [Ca^2+^]_Lumen_ that induces TMCO1 oligomerization (39, 177).

#### 3.1.4. Trimeric intracellular cation channels.

Trimeric intracellular cation channels (TRICs) are localized on the ER/SR membranes (48, 97, 178) (FIGURE 4). There are two isoforms in mammals, TRIC-A and TRIC-B, encoded by *TMEM38A* and *TMEM38B*, respectively (178). Whereas TRIC-A is expressed predominantly in muscle cells, TRIC-B is ubiquitously expressed (96). TRICs are proposed to function as trimetric 7-TM channels (47, 95, 179, 180). In lipid bilayer recordings, TRIC-A and TRIC-B were shown to be voltage-modulated monovalent-selective cation channels with no significant permeability to Ca^2+^ (95, 178, 181), but the significance of the voltage dependence is unclear, as global *E*_K_ and Δψ across the ER membrane are thought to be ∼0 mV.

The channel activities of TRIC-A and TRIC-B are both modulated by Ca^2+^ and lipids (181, 182). Ca^2+^ regulates TRICs from both the cytosolic and luminal sides: whereas cytosolic Ca^2+^ activates, luminal Ca^2+^ inhibits TRICs (95, 181). A low-specificity cation-binding site was proposed to confer cytosolic Ca^2+^ activation (47). Another Ca^2+^-binding site facing the lumen was revealed in frog TRIC-B and chicken TRIC-A and likely mediates the inhibitory effect of high luminal Ca^2+^ (95, 174). The opposite effects of luminal versus cytosolic Ca^2+^ are consistent with the role of TRICs in promoting Ca^2+^ store filling. When the Ca^2+^ store is full, TRICs are kept in an inactive state; store depletion may readily activate the channel (183). One attractive hypothesis is that TRICs may provide counterions, e.g., K^+^ influx, to promote ER Ca^2+^ release through charge compensation or osmolarity maintenance (96, 184) (FIGURE 4). Notably, the large single-channel conductance of IP3Rs and RyRs suggests that Ca^2+^ efflux through these Ca^2+^ channels may cause localized changes in both *E*_Ca_ and Δψ. Therefore, although global *E*_K_ and Δψ are ∼0 mV, the opening of IP3Rs and RyRs may result in a localized cytosolic side-positive Δψ (hyperpolarized ER Δψ) to drive local K^+^ influx by increasing the local electrochemical K^+^ gradient (95) (FIGURE 4). It was proposed that the physiological function of TRICs is to shape Ca^2+^ signals, likely through conducting K^+^ as the counterion to facilitate ER Ca^2+^ release (96, 183, 184). TRICs have also been shown to be modulated by lipids, such as phosphatidylinositol 4,5-bisphosphate [PI(4,5)P_2_] and diacylglycerol (DAG), based on lipid bilayer reconstitution studies (95, 180). However, it is not clear whether these lipids are present in abundance on the ER membrane. Therefore, it is necessary to study the lipid regulation of TRICs in their native ER membranes with the yet-to-be-developed whole ER recordings.

#### 3.1.5. ER Chloride channels.

Chloride Channel CLIC Like 1 (CLCC1), a.k.a. ER Anion Channel 1 or ERAC1, was recently identified as an ER-resident membrane protein that mediates a Cl^−^ conductance based on bilayer electrophysiology (108) (FIGURE 4). Like TRICs, the channel activity of CLCC1 is also inhibited by high luminal Ca^2+^ but activated by PI(4,5)P_2_. Hence, when the store is full CLCC1/ERAC1 channels are kept in an inactive state; a decrease in [Ca^2+^]_ER_ may readily activate the channels (108). In other words, CLCC1 is secondarily activated upon stimulation of ER Ca^2+^-release channels. A localized luminal side-negative Δψ due to local IP3R/RyR openings may drive local Cl^−^ efflux through CLCC1/ERAC1 (108). In CLCC1 KO cells, ER tubules are osmotically enlarged with changes in [Cl^−^]_ER_, [K^+^]_ER_, and [Ca^2+^]_ER_, as revealed by luminal ion imaging. Hence, CLCC1/ERAC1 may also provide counteranions to facilitate ER Ca^2+^ release (108).

### 3.2. Ion Channels of the Golgi Apparatus

Secretory pathway proteins that are synthesized in the ER need to be processed in the Golgi apparatus, e.g., glycosylated, before they are sorted to secretory vesicles for delivery to their destination, e.g., the plasma membrane (27, 185). The Golgi apparatus consists of multiple cisternae, which are a series of stacked membranes and associated vesicles on the *cis*, medial, and *trans* sides (27, 185). Based on their functions and locations, the Golgi apparatus can be separated into several groups: *cis*-Golgi, medial Golgi, and the *trans*-Golgi network (TGN) (27, 94, 185). Whereas *cis*-Golgi is responsible for receiving biosynthetic output from the ER and initial processing by luminal enzymes, the TGN is responsible for further cargo modification and sorting of the secretory pathway proteins to their final destinations through secretory vesicles/granules (see FIGURE 5). Both *E*_Na_ and *E*_K_ across the Golgi membrane are posited to be ∼0 mV, resembling the ER. However, unlike the ER, the TGN may have a negative Δψ that can be utilized to drive ion transport and release (106), including Na^+^/K^+^ flux. The reported TGN Δψ values range from −30 mV to −100 mV (71, 76, 106). However, as TGN *E*_Na_, *E*_K_, and *E*_Cl_ are all close to 0 mV and *E*_H_ is greater than +50 mV, the ionic mechanisms that contribute to very negative Δψ are unclear. Hence, for the sake of discussion, we use the modest Δψ of −30 mV in most contexts of the present review. A recent study showed that heterologously expressed voltage-gated K^+^ channel K_V_11.1 in the TGN may regulate luminal [K^+^], and possibly TGN Δψ (70, 71).

The Golgi apparatus is also believed to serve as an acidic Ca^2+^ store, although [Ca^2+^] and pH are different in the lumens of *cis*-Golgi versus TGN (27, 80, 186) (FIGURE 1 AND FIGURE 5). Like [Ca^2+^]_ER_, [Ca^2+^]_TGN_ and [Ca^2+^]*_cis_*_-Golgi_ are mainly maintained and balanced by SERCA and IP3Rs/RyRs (186–189) (FIGURE 5). In addition, SPCAs (secretory pathway calcium ATPases) also function as TGN Ca^2+^ pumps, although they also transport other divalent ions such as Mn^2+^ (190). With the use of luminally localized bioluminescent reporters, [Ca^2+^]_TGN_ is estimated to be ∼0.1–0.3 mM (TGN *E*_Ca_ greater than +150 mV) (186). Besides IP3Rs/RyRs, transmembrane BAX inhibitor motif (TMBIM) proteins were also proposed to mediate Ca^2+^ efflux from the Golgi apparatus (191, 192), although recent studies suggested that some members of this protein family are lysosomal Ca^2+^ channels (192–194) or regulators of lysosomal Ca^2+^ stores (194). Besides a role in signal transduction such as that of ER Ca^2+^, Ca^2+^ in the Golgi apparatus may regulate both luminal functions, e.g., glycosylation and sorting, and membrane trafficking (27, 42, 195). However, the specific Golgi Ca^2+^ channel(s) that regulates Ca^2+^-dependent membrane fusion of Golgi-derived vesicles is not known.

The lumen of the Golgi apparatus is acidic, with a small gradient in the luminal pH from *cis*-Golgi (pH 6.6, *E*_H_ greater than +50 mV) to TGN (pH 6.0, *E*_H_ greater than +80 mV) (30, 94) (see FIGURE 1 AND FIGURE 5). The acidic lumen of TGN is established and maintained by the V-ATPase (30, 94). To support sustained electrogenic V-ATPase pumping, counterion conductances are necessary. ClC-3b, the Golgi-localized ClC-3 variant, and GPHR (Golgi pH regulator), a proposed voltage-dependent anion channel, were reported to mediate the counteranion currents (196, 197) (FIGURE 5). However, because TGN *E*_Cl_ is close to TGN Δψ, a driving force for Cl^−^ efflux will be created when the modest TGN Δψ is temporarily decreased (less negative) or reversed, e.g., upon localized Ca^2+^ efflux. As inhibition of V-ATPase leads to Golgi deacidification, a “H^+^ leak” conductance may exist on the TGN membrane (94). However, its molecular identity remains elusive (94). A recent study suggested that STING may act as a cyclic GMP-AMP synthase (cGAS)-activated H^+^ channel/transporter on the Golgi membrane (198).

### 3.3. Ion Channels of Secretory Vesicles and Secretory Granules

As secretory vesicles are derived from TGN, they have similar ionic compositions and membrane channels/transporters, e.g., IP3Rs and RyRs (94, 199). However, although the secretory vesicles are also acidic Ca^2+^ stores, their [Ca^2+^]_Lumen_ and pH_Lumen_ are slightly lower (27, 80, 94, 186) (see FIGURE 1). In addition, sorting of membrane proteins occurs from *cis*-Golgi to TGN and from TGN to the secretory vesicles (80, 186) (see FIGURE 5). Various ectopically expressed TRP channels, as well as other plasma membrane channels, were found to be localized on the secretory vesicles (78). However, it is not clear whether they are resident channels in the secretory vesicles, i.e., in the “driver’s seat,” or just trafficking cargo proteins that are only temporarily localized on the secretory vesicles during the secretory pathway, i.e., in the “passenger seat” (56, 78). It is generally believed that plasmalemmal channels are not active in their secretory pathway, because of the lack of plasma membrane-specific lipids, e.g., PI(4,5)P_2_ (78). Because secretory vesicles are undergoing constant traffic, it is expected that cargo proteins in the secretory vesicles have a short “dwell time,” whereas the resident membrane proteins in the secretory vesicle have a long dwell time. However, in the overexpression experiments, the dwell time may be extended. Hence, it is necessary to develop functional assays, e.g., whole secretory vesicle recordings, to test the functionality of the ion channels of secretory vesicles. Secretory vesicles in specialized cell types, i.e., secretory granules, may be equipped with various organellar channels/transporters. For instance, plasma membrane store-operated Orai channels are localized in secretory granules of the neurosecretory cells, mediating store-operated Ca^2+^ release from secretory granules upon receptor stimulation (200). N-type calcium channels are detected in the secretory granules of PC12 and chromaffin cells (201). Likewise, CLCA, a reported Ca^2+^-activated anion channel, and CLC-2, a member of the CLC family of Cl^−^ channels/transporters, are also expressed on the acinar zymogen granules (199). However, direct electrophysiological studies of granular membranes are lacking, so it is not clear whether the channels are functionally active in these vesicles as noncommitted organellar channels. It is important to note that a bilayer recording is insufficient to prove that a noncommitted organellar channel is operative physiologically in secretory vesicles/granules.

## 4. ION CHANNELS IN THE DEGRADATION PATHWAY

### 4.1. Endosome Maturation, Autophagy, and Lysosome Degradation

Lysosomes are the cell’s degradation center, primarily responsible for the breakdown of various cargo materials such as proteins, polysaccharides, and complex lipids into their respective building-block molecules: amino acids, monosaccharides, and free fatty acids (7, 8). Extracellular cargo materials are delivered to lysosomes through endocytosis and phagocytosis (8). Upon endocytosis, cargo materials are sorted first to early endosomes and then to late endosomes, which are gradually acidified by the V-ATPase (29) (FIGURE 1). In addition, luminal Ca^2+^ concentrations also change significantly during endosome maturation (FIGURE 1). H^+^ and Ca^2+^ flux across endosomal membranes may regulate membrane trafficking and the speed of endosome maturation (28, 42, 77). Intracellular cargo materials, e.g., damaged mitochondria and misfolded protein aggregates, are packed into autophagosomes and then delivered to lysosomes for degradation (202). H^+^ and Ca^2+^ flux across autophagosomal and lysosomal membranes may regulate autophagosome formation and maturation (73, 86, 203–205). Lysosomal Ca^2+^ is thought to regulate both autophagosome-lysosome fusion and late endosome-lysosome fusion (13, 206).

Catabolic degradation is mediated by >60 different types of hydrolases, including proteases, lipases, and glycosidases (207). The degradation products, i.e., catabolites, are exported out of lysosomes for reutilization in the biosynthetic pathways through Na^+^/H^+^-dependent transporters (36, 107). Alternatively, some catabolites are exported through vesicular membrane trafficking or nonvesicular membrane contact mechanisms (5, 13, 60). Many parameters of lysosomes, e.g., number, location, size, shape, and activity, are regulated by nutrient status and cellular signaling (13, 23, 208). Defective degradation, catabolite export, or trafficking leads to lysosomal dysfunction and LSDs (23).

Ion channels on the lysosomal membrane “bridge” the lysosomal lumen, where the primary function of the lysosome (i.e., degradation) occurs, and the cytosol, where the signaling that regulates degradation takes place (13, 25) (FIGURE 3). First, lysosome function requires the maintenance of luminal homeostasis, especially ionic homeostasis and membrane potential stability (13, 207). For example, most lysosomal hydrolases require an acidic and high-Cl^−^ lumen to function (29, 53, 209). H^+^ pumping for lysosomal acidification is also dependent on the lysosomal Δψ, which is determined by various ionic permeabilities in the lysosomes (13, 98, 100, 210). In addition, V-ATPase-dependent lysosomal acidification also requires the efflux of countercations and the influx of counteranions (98, 100). Second, many catabolite exporters are sensitive to lysosomal Δψ, suggesting that lysosomal ion channels may also regulate the export of degradation products (36, 211). Third, lysosomal trafficking is regulated by H^+^ homeostasis, Δψ, and Ca^2+^ (28, 51, 62). Although H^+^ flux and Δψ may also indirectly affect lysosomal Ca^2+^ release (46, 212, 213), Ca^2+^ is known to regulate most steps in lysosomal trafficking, including the fusion of lysosomes with autophagosomes and late endosomes (7, 26, 214) (FIGURE 3).

### 4.2. Ion Channels in the Late Endosomes and (Endo-, Auto-, and Phago-) Lysosomes

#### 4.2.1. TRPML1.

TRPML1 (a.k.a MCOLN1) is the major Ca^2+^-permeable channel in the LELs of all cell types in mammals (44). TRPML1 was independently cloned by three groups as the product of the gene underlying type IV mucolipidosis (ML-IV), a neurodegenerative LSD (215–217). Like other TRP channels, TRPML1 consists of six transmembrane domains (TMs; S1–S6) with the NH_2_ and COOH termini facing the cytosol (218, 219). TRPML1 is primarily localized and indeed highly enriched in the LELs but not in other endomembrane organelles, suggesting that TRPML1 is a committed lysosomal channel (44). Although overexpressed TRPML1 proteins may also be present at the plasma membrane, in the endogenous setting two double-leucine motifs direct TRPML1 proteins to LELs (62, 220).

##### 4.2.1.1. channel permeation and selectivity.

The LEL localization of TRPML1 made it challenging to analyze the permeation and gating properties of the channel. However, the development of the whole LEL patch-clamp technique allowed the direct study of TRPML1 on artificially enlarged LELs (33, 51, 120). With the use of whole LEL patch-clamp recording (FIGURE 2*A*), it was shown that TRPML1-mediated currents exhibit strong inward rectification (inward indicates cations moving out of the lysosomal lumen) (FIGURE 3) (44, 120, 133). TRPML1 is permeable to Ca^2+^, Na^+^, K^+^, Fe^2+^, and Zn^2+^ but not to H^+^ (33, 133, 221) (TABLE 1). The selectivity filter of TRPML1 is formed by the “pore-loop” region between S5 and S6 (218, 219). Pore mutations of TRPML1 are known to affect the permeation of TRPML1 channels: for example, replacing two negatively charged amino acid residues in the pore loop with positively charged ones (D^471^D^472^-KK) results in a nonconducting pore-dead channel (120, 220–223). By employing lysosome-targeted GECIs (e.g., GCaMP-TRPML1; see FIGURE 2*B*) to measure juxtalysosomal Ca^2+^, it was found that TRPML1 mediates Ca^2+^ release from LELs in intact cells (46, 62, 85). With the use of lysosome-targeted voltage indicators, it was shown that activation of TRPML1 causes lysosomal depolarization (i.e., reducing Δψ) (106), consistent with the positive values of lysosomal *E*_Na_ and *E*_Ca_.

**Table 1.** Summary of organellar channels

Name	Subcellular Distribution	Permeability/Selectivity	Endogenous Agonists	Synthetic Agonists	Endogenous Inhibitors	Synthetic Inhibitors
*Committed organellar channels*						
IP_3_Rs	ER, Golgi	Ca^2+^, K^+^, Na^+^	IP_3_, ATP, cytosolic Ca^2+^ (low)	Adenophostin A	Cytosolic Ca^2+^ (high), luminal Ca^2+^	2-APB, decavanadate, xestospongin C
RyRs	ER, Golgi	Ca^2+^, K^+^, Na^+^	Cytosolic Ca^2+^ (low), ATP, luminal Ca^2+^	Caffeine, suramin, ryanodine (nanomolar to micromolar range)	Cytosolic Ca^2+^ (high), Cytosolic Mg^2+^	Ryanodine, dantrolene, procaine
TRIC-A/B	ER	K^+^, Na^+^	Δψ		Luminal Ca^2+^	
TMCO1	ER	K^+^, Ca^2+^	High luminal Ca^2+^			
CLCC1	ER	Br^−^, Cl^−^, $NO_{3}^{-}$, F^−^	PI(4,5)P_2_		Luminal Ca^2+^	DIDS
TMBIM1–4	Golgi	Na^+^, K^+^, Ca^2+^			H^+^	
GPHR	Golgi	Cl^−^, Br^−^, I^−^, F^−^	Δψ		DIDS	
TRPML1	LEL, TV	Ca^2+^, Na^+^, K^+^, Zn^2+^, and Fe^2+^	PI(3,5)P_2_, ROS	ML-SAs, SF51, MK-683, ML1-SA1	PI(4,5)P_2_, sphingomyelin, adenosine	ML-SIs, estradiol analogs (EMDE, PRU-10, PRU-12)
TRPML2	LEL, RE	Ca^2+^, Na^+^, K^+^, Fe^2+^	PI(3,5)P_2_, ROS	ML-SAs, ML2-SA1, SFs		ML-SIs
TRPML3	LEL, EE, autophagosome	Ca^2+^, Na^+^, K^+^	PI(3)P_,_ PI(3,5)P_2_	ML-SAs, ML3-SA1, SFs	Luminal H^+^, PI(4,5)P_2_	ML-SIs
TPC1	LEL, macropinosome, EE	Na^+^, Ca^2+^	PI(3)P, PI(3,5)P_2_, Δψ, sphingosine (?), NAADP (?)	Clomipramine	ATP/mTOR, luminal H^+^	Verapamil, tetrandrine (?), Ned19 (?)
TPC2	LEL, melanosome	Na^+^, Ca^2+^	PI(3,5)P_2_, NAADP (?)	Riluzole, clomipramine, TPC2-A1-P, and TPC2-A1-N	ATP/mTOR, Mg^2+^	Verapamil, tetrandrine (?), Ned19 (?), SG-005, SG-094
TMEM175	LEL, EE	H^+^, K^+^, and Cs^+^	Luminal H^+^, ArA	DCPIB, ML67-33		4-AP
CLN7	LEL	Cl^−^, I^−^, F^−^				DIDS, NPPB
OCA2	Melanosome	Cl^−^, Br^−^, I^−^, F^−^				
*Noncommitted organellar channels*						
Lyso-BK (SLO1)	PM, LEL, nucleus	K^+^	Ca^2+^	NS 1619		Paxiline, IBTX
Lyso-VRAC (LRRC8A)	PM, LEL	Cl^−^, I^−^, $NO_{3}^{-}$	Low Г			DCPIB, NS 3728
P2X4	PM, LEL	Ca^2+^, Na^+^, K^+^	Luminal ATP		H^+^	
TWIK2	PM, LEL	K^+^				Quinidine
PAC (TMEM206)	PM, EE, macropinosome	Cl^−^, Br^−^, I^−^, $NO_{3}^{-}$, SCN^−^	H^+^		PI(3,5)P_2_, PI(4,5)P_2_, PI(3,4,5)P_3_	DIDS, NPPB, NFA

ArA, arachidonic acid; DCPIB, 4-[(2-butyl-6,7-dichloro-2-cyclopentyl-2,3-dihydro-1-oxo-1*H*-inden-5-yl)oxy]butanoic acid; DIDS, 4,4′-diisothiocyano-2,2′-stilbenedisulfonic acid; EE, early endosome; IBTX, iberiotoxin; IP_3_, inositol (1,4,5)-trisphosphate; IP3R, IP_3_ receptor; LEL, late endosome and lysosome; ML 67-33, 2,7-dichloro-9,10-dihydro-9,9-dimethyl-10-[2-(2*H*-tetrazol-5-yl)ethyl]acridine; ML-SAs, mucolipin synthetic agonists; ML-SIs, mucolipin synthetic inhibitors; NAADP, nicotinic acid adenine dinucleotide phosphate; NFA, niflumic acid; NPPB, 5-nitro-2-(3-phenylpropylamino)benzoic acid; PI(3)P, phosphatidylinositol 3-phosphate; PI(3,5)P_2_, phosphatidylinositol 3,5-bisphosphate; PI(4,5)P_2_, phosphatidylinositol 4,5-bisphosphate; PM, plasma membrane; RE, recycling endosome; ROS, reactive oxygen species; RyR, ryanodine receptor; TRIC, trimeric intracellular cation channel; TV, tubulovesicle; 4-AP, 4-aminopyridine; Г, ionic strength.

##### 4.2.1.2. channel modulation.

###### 4.2.1.2.1. Luminal protons.

Lysosomal channels are believed to encode various lysosome-acting cellular cues that regulate lysosomal functions (13). For instance, several lysosomal channels are sensitive to low luminal pH (pH_Ly_); TRPML1 currents are potentiated by low pH_Ly_ (33, 221, 224). However, pH_Ly_ has been reported to increase the monovalent conductance of TRPML1 but not Ca^2+^ flux (13, 221). Hence, cellular cues affecting lysosome acidification may regulate lysosomal functions via TRPML-dependent mechanisms. Structural analysis revealed that H^+^ interacts with the luminal pore domain of TRPML1 to regulate its cation conductance (224).

###### 4.2.1.2.2. Phosphoinositides.

**FIGURE 6.**
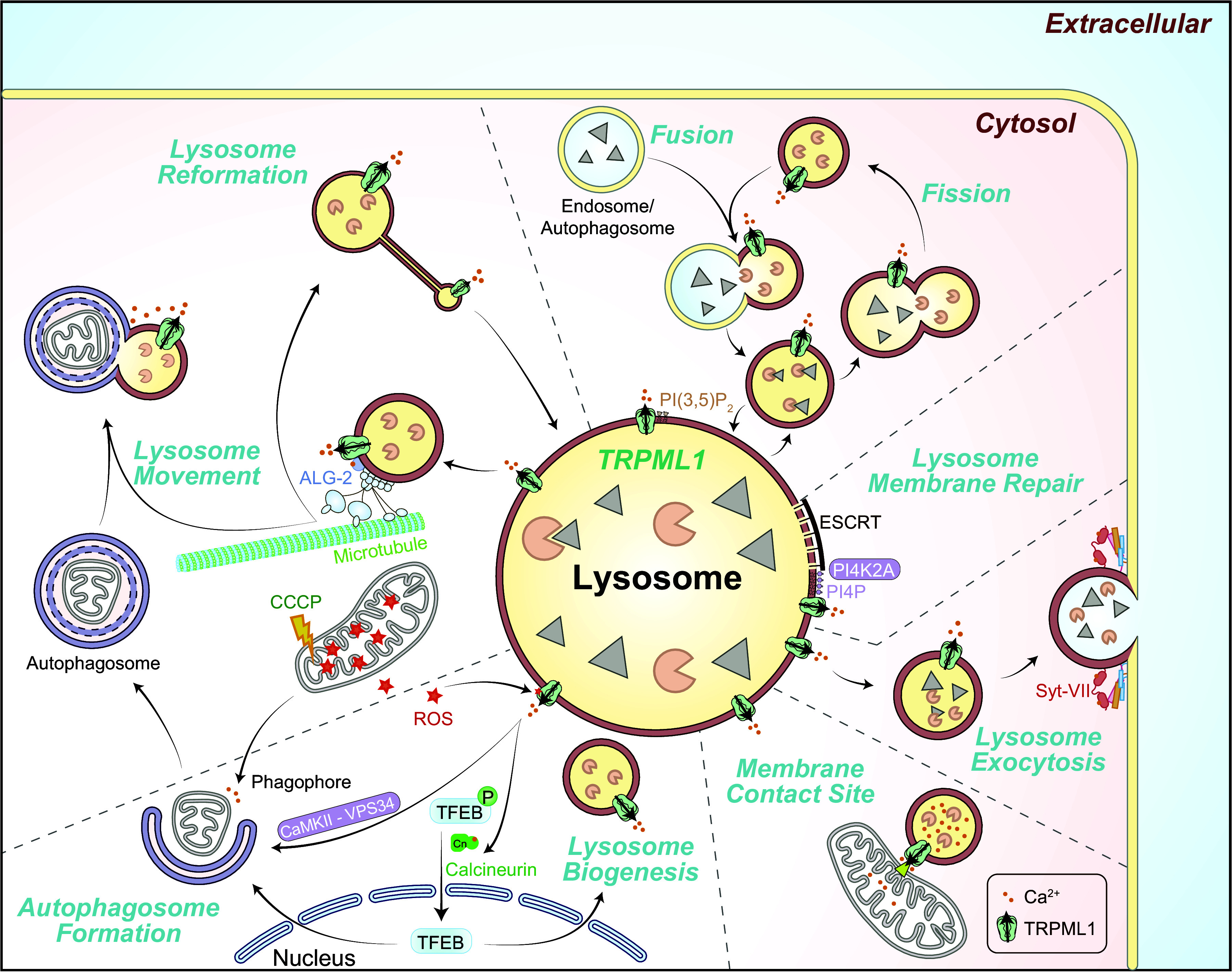
The cell biological functions of mucolipin TRP channel (TRPML)1. TRPML1-mediated Ca^2+^ release from lysosomes regulates multiple lysosomal functions: *1*) fusion of lysosomes with autophagosomes, late endosomes, and phagosomes; *2*) fission from endolysosomes, autolysosomes, and phagolysosomes; *3*) retrograde transport of lysosomes along microtubules via a phosphatidylinositol 3,5-bisphosphate [PI(3,5)P_2_]-TRPML1-ALG2-dynein pathway; *4*) autophagosome formation via a CaMKII-VPS34 pathway; *5*) lysosomal exocytosis using the Syt-VII Ca^2+^ sensor; *6*) endosomal sorting complex required for transport (ESCRT)/phosphatidylinositol 4-phosphate (PI4P)-mediated lysosomal membrane repair; *7*) lysosomal reformation. Upon completion of lysosomal degradation, lysosomes undergo extensive tubulation to become tubular lysosomes, from which protolysosomes are regenerated via a fission-based “budding-off” mechanism, *8*) promoting the formation of lysosome-mitochondria membrane contact sites and *9*) activation (i.e., nuclear translocation) of transcription factor EB (TFEB), a transcriptional factor and master regulator of lysosome biogenesis and autophagosome biogenesis. Upon TRPML1-mediated lysosomal Ca^2+^ release, activated calcineurin dephosphorylates TFEB, which can translocate to the nucleus, initiating the expression of lysosomal and autophagic genes. Hence, the TRPML1-Ca^2+^-TFEB pathway is a key regulator of lysosome biogenesis and autophagy. ROS, reactive oxygen species.

TRPML1 channels are activated in a low-nanomolar range by phosphatidylinositol 3,5-bisphosphate [PI(3,5)P_2_], a LEL-specific phosphoinositide that regulates many aspects of lysosomal trafficking (120, 219, 225) (FIGURE 3 AND FIGURE 6; TABLE 1). PI(3,5)P_2_ binds to positively charged amino acid residues in the cytosolic NH_2_-terminal region of the channel as well as the cytosolic ends of the S1 and S2 helices (225), resulting in the opening of the S6 gate through the S2–S3 linker (219, 226, 227). Whereas cellular PI(3,5)P_2_ levels are shown to change before lysosomal trafficking events, in PI(3,5)P_2_-deficient cells many lysosomal functions are defective, including lysosomal retrograde movement, exocytosis, and reformation (25, 228–230). Hence, TRPML1 may serve as an essential signal transducer for lysosomal PI(3,5)P_2_. Consistently, mutations in the PI(3,5)P_2_ binding sites of TRPML1 affect PI(3,5)P_2_-dependent lysosomal functions, causing lysosomal trafficking defects mimicking ML-IV cells (89, 230). However, other PI(3,5)P_2_ effectors may also contribute to the regulation of these lysosomal functions (230).

PI(4,5)P_2_, a cell surface phosphoinositide, was shown to inhibit TRPML1 (TABLE 1), and such inhibition is proposed to prevent TRPML1 from being active in nonlysosomal compartments (121, 231). However, PI(4,5)P_2_ was reported to be generated on the lysosomal membrane to regulate mTOR-dependent lysosome reformation (232). As lysosome reformation is a Ca^2+^-dependent process (89), it is possible that PI(4,5)P_2_ inhibition of TRPML1 plays an important role in lysosomal reformation. Lysosomal PI(4,5)P_2_ levels are aberrantly elevated in some LSD cells (233). Hence, pathogenic inhibition of TRPML1 may underlie the trafficking defects in some LSDs. Collectively, compartment-specific regulation of TRPML1 by phosphoinositides may be responsible for several TRPML1-mediated lysosomal functions in both physiology and pathophysiology.

###### 4.2.1.2.3. Reactive oxygen species.

Reactive oxygen species (ROS) are environmental stress signals that regulate a number of cellular functions, including autophagosome and lysosome biogenesis (234, 235). ROS levels are elevated upon mitochondrial damage, and this triggers mitophagy to remove damaged mitochondria and excessive ROS as a negative-feedback mechanism to maintain cellular health (234). ROS induce nuclear translocation of the transcription factor EB (TFEB), a master regulator of autophagosome and lysosome biogenesis (59), in a TRPML1- and lysosomal Ca^2+^-dependent manner (234). ROS directly and robustly activate lysosomal TRPML1 channels, suggesting that TRPML1 may function as a signal transducer for ROS to regulate lysosome function (234, 235). Consistent with this hypothesis, the ROS sensitivity of TRPML1 is shown to be required for ROS-induced TFEB activation and mitophagy (234). Generally speaking, because lysosomal channels like TRPML1 are activated by more than one cellular cue, to test which activation mechanism is key to a specific function it is necessary to introduce knockin mutations at agonist-specific binding sites (13).

###### 4.2.1.2.4. Lipidated LC3 proteins.

Lysosome damage leads to TFEB nuclear translocation to initiate lysosome biogenesis (111). TRPML1, by interacting directly with lipidated LC3, a microtubule-associated protein light chain 3 that is recruited to the lysosomal outer surface after lysosomal damage, mediates lysosome Ca^2+^ release to trigger TFEB activation (236, 237). However, whether lipidated LC3 can act as an endogenous activator of TRPML1 remains unclear.

###### 4.2.1.2.5. Adenosine and sphingomyelins.

Defective degradation may cause substrate accumulation in lysosomes to indirectly affect TRPML1 activity, which may trigger a vicious cycle between “degradation” and “trafficking block” (25, 62). Pathological accumulation of adenosine and sphingomyelins in the LELs was shown to inhibit TRPML1 (62, 238). Adenosine is produced by adenosine deaminase (ADA) catalysis in lysosomes, and ADA dysfunction leads to ML-IV-like phenotypes caused by adenosine inhibition of TRPML1 (238). Likewise, in Niemann–Pick disease types A, B, and C, luminal sphingomyelin accumulation due to the reduced acid sphingomyelinase activity may inhibit TRPML1, causing lysosomal dysfunction, trafficking defects, and LSD phenotypes (62, 239).

##### 4.2.1.3. natural/synthetic agonists and inhibitors.

###### 4.2.1.3.1. Rapamycin.

Rapamycin, a natural compound that is widely known as an inhibitor of mTOR, was found unexpectedly to activate TRPML1 as a partial agonist, independent of its effects on mTOR (240) (TABLE 1). Direct binding of rapamycin to TRPML1 was confirmed in cryo-electron microscopy (cryo-EM) costructures (225, 240). Rapamycin is well known for its antiaging and neuroprotective effects (225, 240). Because rapamycin has effects on both mTOR and TRPML1, it is necessary to introduce mutations in the rapamycin-binding site of TRPML1 and investigate whether TRPML1 activation is primarily responsible for the antiaging and neuroprotective effects of rapamycin.

###### 4.2.1.3.2. TRPML-specific synthetic agonists and antagonists.

To probe TRPML-dependent lysosomal functions, small-molecule synthetic modulators have been identified via Ca^2+^ imaging-based high-throughput screening (HTS) (62, 228, 241). TRPML-specific synthetic agonists, e.g., ML-SA1, by binding to a hydrophobic pocket located above the S5–S6 gate in cryo-EM costructures, specifically activate lysosomal TRPML channels but not other lysosomal ion channels (218, 242, 243) (FIGURE 3 and TABLE 1). MK6-83, a related compound, was shown to restore endolysosomal trafficking defects in ML-IV fibroblasts caused by hypofunctional mutations of TRPML1 (62, 241, 244). ML-SA1-binding mutations were reported to selectively abolish ML-SA1 activation without affecting PI(3,5)P_2_ activation (218). Hence, synthetic agonists may provide a powerful tool to activate TRPML1, linking the channel activity with specific lysosomal functions. In macrophages, acute ML-SA1 treatment induces lysosomal exocytosis (as assayed by LAMP1 surface staining) mediated by lysosomal Ca^2+^ sensor Syt-VII (90) in wild-type (WT) but not TRPML1 KO cells (88) (FIGURE 6). Moreover, retrograde movement of lysosomes to the perinuclear region, which is required for autophagosome-lysosome fusion and lysosomal tubulation, is increased with TRPML1 overexpression or synthetic agonists through Ca^2+^ sensor EF-hand protein ALG2 but reduced by TRPML1 KO or PI(3,5)P_2_ deficiency (89) (FIGURE 6). Hence, activation of TRPML1 may allow cellular cues such as lysosomal lipids and synthetic agonists to regulate lysosomal trafficking by triggering increases in juxtalysosomal Ca^2+^ levels. The first generation of ML-SA compounds are all pan-TRPML agonists. However, relatively specific synthetic agonists of TRPML1, e.g., ML1-SA1, have now been developed (61). TRPML-specific synthetic inhibitor (ML-SI) compounds were also identified through Ca^2+^-imaging-based HTS (88) (TABLE 1). ML-SIs share the same binding pocket with ML-SA1, as evidenced by cryo-EM structural analysis (245), and have been used to demonstrate the essential roles of TRPML1 in regulating various lysosomal functions (88, 89, 234). Recently, several estradiol analogs, e.g., EMDE, PRU-10, and PRU-12, were shown to inhibit TRPML1’s channel activity and TRPML1-dependent cellular functions (246) (TABLE 1).

##### 4.2.1.4. cell biological functions.

To mediate degradation, lysosomes must undergo extensive membrane trafficking, including movement along microtubule tracks toward cargo-carrying endosomes and autophagosomes and subsequent membrane fusion/fission. A general challenge for both membrane trafficking and LSD studies is that gene manipulations may cause cellular phenotypes through indirect mechanisms. Hence, manipulating TRPML1 activity acutely with synthetic agonists (ML-SAs) and antagonists (ML-SIs), together with genetic methods (e.g., knockouts as negative controls), may prove uniquely advantageous in identifying the direct effects. For example, in lysosome (chemical) biology studies, by acutely manipulating the activity of TRPML channels it was demonstrated that TRPMLs play a key role in lysosomal exocytosis and that TRPML-dependent lysosomal exocytosis is in turn required for many basic cellular processes, including large-particle phagocytosis (88), membrane repair (243), and pathogen expulsion (247).

###### 4.2.1.4.1. Lysosomal Ca^2+^ release.

TRPML1 is primarily localized in LELs, mediating lysosomal Ca^2+^ release in response to cellular cues (13, 25, 56). With the use of a lysosome-targeted GECI (GCaMP-TRPML1; see FIGURE 2*B*), it was shown that activation of TRPML1 by ML-SAs is sufficient to cause lysosomal Ca^2+^ release (56, 62, 85, 248). On the other hand, lysosomal Ca^2+^ release in response to starvation is reduced when TRPML1 is genetically inactivated or pharmacologically inhibited (88, 212). Downstream of TRPML1, various Ca^2+^ sensors such as Syt-VII, calmodulin, and ALG-2 might be activated to regulate cue-specific lysosomal trafficking steps (89, 91, 243, 249).

###### 4.2.1.4.2. Lysosomal Fe^2+^/Zn^2+^ release.

TRPML1 is permeable to Fe^2+^ and Zn^2+^ in addition to Ca^2+^ (33). TRPML1 was thus proposed to mediate Fe^2+^ and Zn^2+^ transport across lysosomal membranes (33, 112–114), providing iron/zinc to the cytosol for cellular metabolism (112). Cells lacking TRPML1 exhibit a cytosolic Fe^2+^ deficiency and an overload of lysosomal Fe^2+^, suggesting that TRPML1 contributes to Fe^2+^ transport out of the lysosomes (33). Consistently, activation of TRPML1 promotes iron-induced cell death (250). Likewise, lysosomal Zn^2+^ accumulation in TRPML1 KO cells is also suggestive of a role of TRPML1 in lysosomal Zn^2+^ transport (251). Consistently, ML-SA activation of TRPML1 induces lysosomal Zn^2+^ release (113). In metastatic melanoma cells, in which TRPML1 is pathologically upregulated, activation of TRPML1-dependent lysosomal Zn^2+^ release may trigger mitochondrion-dependent necrotic cell death (114). The Zn^2+^ effectors in these processes are not known.

###### 4.2.1.4.3. Lysosomal exocytosis.

The fusion of lysosomes with the plasma membrane, i.e., lysosomal exocytosis, is a Ca^2+^-dependent process (252). Lysosomal exocytosis is impaired in ML-IV patient cells (253, 254), and activation of TRPML1 enhances lysosomal exocytosis (64, 88, 133) (see FIGURE 6). Syt-VII is likely the Ca^2+^ sensor for lysosomal exocytosis, as dominant-negative Syt-VII was shown to inhibit lysosome exocytosis (88, 90, 115). Macrophages use TRPML1-dependent lysosomal exocytosis to supply lysosomal membranes to the plasma membrane during large-particle phagocytosis (88). In muscle cells, TRPML1-dependent lysosomal exocytosis can promote plasma membrane resealing by supplying additional membranes (243, 249). TRPML1 is required for cellular clearance induced by TFEB overexpression in most LSD cells, except ML-IV, suggesting that TRPML1-dependent lysosomal exocytosis may help remove damaged or unwanted cellular materials (64, 255).

###### 4.2.1.4.4. Lysosomal membrane repair.

When lysosomes are osmotically or chemically damaged, a lysosomal membrane repair response is triggered (111, 256). Activation of TRPML1 by ML-SA1 is sufficient to trigger the lysosomal repair pathway (111, 256) (FIGURE 6). Upon lysosomal damage, lipidated LC3 is recruited to the lysosomes, interacting with TRPML1 to release Ca^2+^ (236). Hence, LC3 lipidation might activate lysosomal TRPML1 channels during lysosomal membrane damage, but direct evidence is still lacking.

###### 4.2.1.4.5. Lysosomal membrane fusion.

Lysosomal Ca^2+^ release may promote the fusion of lysosomes with late endosomes, autophagosomes, and phagosomes, which receive cargo materials from the endocytic, autophagic, and phagocytic pathways (233, 257). There are three sequential steps during membrane fusion: tethering, SNARE complex formation, and lipid bilayer mixing (7, 258). Lysosomal Ca^2+^ release may regulate one or more fusion steps, similar to Ca^2+^ regulation of synaptic vesicle exocytosis (13, 91, 257). TRPML1 is proposed to regulate Ca^2+^-dependent lysosomal fusion with late endosomes, autophagosomes (13, 204, 259, 260), and phagosomes (257, 261) (see FIGURE 6). Lysosomal PI(3,5)P_2_ level increases before the fusion events of lysosomes (88, 228), suggesting that PI(3,5)P_2_ may be an endogenous agonist of TRPML1 for triggering lysosomal fusion. When TRPML1 is inhibited, e.g., when TRPML1 inhibitor PI(4,5)P_2_ is pathologically accumulated in the lysosomes of 5-phosphatase OCRL KO cells, autophagosome-lysosome fusion is blocked (233). Likewise, phagosome-lysosome fusion is blocked in TRPML1 KO macrophages, as well as in WT macrophages when PIKfyve, the PI(3,5)P_2_-synthesizing enzyme, is pharmacologically inhibited (257). Hence, the specificity of lysosomal trafficking events might be determined by the spatial and temporal regulation of PI(3.5)P_2_ levels in the individual lysosomes. However, the mechanism by which PIKfyve activity is regulated by Rab proteins, tethering factors, or membrane curvature is not known.

###### 4.2.1.4.6. Lysosomal membrane fission.

Lysosomal membrane fission can serve two purposes: retrieval/export of degradative products and lysosomal resolution/reformation (7, 262). Additionally, inward budding to form intraluminal vesicles may also regulate membrane remodeling and sorting (263). Lysosome membrane fission is required for retrograde lysosome-to-TGN membrane trafficking, as well as reformation of lysosomes from autolysosomes, phagolysosomes, and endolysosomes (262). Defects in the above-mentioned trafficking steps are observed in TRPML1 KO cells, suggesting a role of TRPML1 in lysosomal membrane fission (13, 89, 91, 228). Consistently, increasing TRPML1’s activity is shown to promote lysosomal membrane fission, and calmodulin is likely the Ca^2+^ sensor (89, 91, 93).

###### 4.2.1.4.7. Lysosome movement and positioning.

For lysosomes to degrade cargos from autophagy and endocytosis, lysosomes must move along the microtubule track toward the cargo-containing autophagosomes in the perinuclear region. A complete pathway (from the signaling molecule to the Ca^2+^ sensor and the motor protein) was identified to regulate on-demand retrograde transport of lysosomes upon autophagy induction: PI(3,5)P_2_-TRPML1-Ca^2+^-ALG-2-dynactin/dynein (89, 93) (FIGURE 6).

###### 4.2.1.4.8. Autophagosome biogenesis.

Autophagy is a process by which cells break down and recycle their own components in response to cellular stressors such as nutrient deprivation or oxidative damage (202, 264). Autophagic dysfunction was found in ML-IV and TRPML1-deficient cells (265). TRPML1-mediated Ca^2+^ release has been proposed to regulate autophagosome biogenesis through promoting the formation of ULK1 and VPS34 complexes via the Ca^2+^-dependent kinase CaMKKβ (204) (FIGURE 6). In addition, activation of TRPML1 may cause nuclear translocation of TFEB, a transcriptional factor and master regulator of autophagosome biogenesis (212, 234). Hence, TRPML1 may regulate autophagosome biogenesis through both transcriptional and posttranslational mechanisms (FIGURE 6). Upon starvation, lysosomal Ca^2+^ release is triggered, in which TRPML1 is involved (86, 212). However, the mechanism by which nutritional cues or the lack thereof activate TRPML1 upon autophagy induction is not clear. One candidate is ROS, as these endogenous agonists of TRPML1 are reportedly elevated under starvation (234). Another candidate is mTOR, which reportedly phosphorylates TRPML1 to inhibit its channel activity (266, 267). A third candidate is lipidated LC3, whose level is increased upon starvation (236).

###### 4.2.1.4.9. TFEB-dependent lysosome biogenesis.

TFEB is a master regulator of lysosome biogenesis (59) and is activated under conditions of cellular stress, including nutrient deprivation, oxidative stress, and infection (63, 212, 250, 268). Upon activation of TRPML1 to trigger lysosomal Ca^2+^ release, it is thought that calcineurin dephosphorylates TFEB, promoting its nuclear translocation (212, 269, 270). Subsequently, activated TFEB increases the expression of lysosomal and autophagic genes (59). Whereas activation of TFEB dramatically upregulates TRPML1’s expression (269), activation of TRPML1, in turn, facilitates calcineurin-dependent dephosphorylation (activation) of TFEB (212) (FIGURE 6). Hence, a positive feedback loop between TFEB and TRPML1 may constitute a quality-control system that regulates lysosome homeostasis according to external cues and intracellular signaling (13, 23). Activation of TFEB and/or TRPML1 is shown to promote mitophagy, which in turn removes damaged mitochondria to reduce excessive ROS in the cell (234). In addition, small-molecule activation of TRPML1 or transgenic overexpression of TRPML1 was found to promote lysosome biogenesis and exocytosis to facilitate membrane repair upon exercise-induced damage in the Duchenne muscular dystrophy (DMD) mouse model (234, 243, 249).

###### 4.2.1.4.10. Lysosome-mitochondria membrane contact.

Ca^2+^ is known to promote or stabilize organelle-organelle MCS formation (5, 10, 11). TRPML1 may contribute to MCS formation between lysosomes and mitochondria (160, 271). Mitochondrial Ca^2+^ level is elevated upon lysosomal TRPML1 activation, and the elevation requires the lysosome-mitochondria MCSs, which facilitate Ca^2+^ transfer from lysosomes to mitochondria (160) (FIGURE 6).

#### 4.2.2. Common lysosomal functions of TRPML1, TRPML2, and TRPML3.

Like TRPML1, TRPML2 and TRPML3 also consist of four identical 6-TM subunits to form a tetrameric channel (227, 242, 272, 273) and are also permeable to Ca^2+^ as well as Na^+^ and K^+^ (33, 274–276) (TABLE 1). In addition, PI(3,5)P_2_ potently activates all three TRPMLs (120, 219, 227). Both TRPML2 and TRPML3 are also localized in LELs, but only in certain cell types (25, 32, 277, 278). In these cell types, TRPML2 and TRPML3 may regulate lysosomal Ca^2+^ release and Ca^2+^-dependent lysosomal trafficking, just like how TRPML1 regulates such processes (73, 203, 279–281). The lysosomal localization of multiple TRPMLs in one cell type suggests that they may play complementary roles in lysosome regulation. For example, whereas TRPML1 and TRPML3 play redundant roles in intestinal enterocytes and cochlear hair cells (282), both TRPML1 and TRPML2 regulate lysosomal functions in B lymphocytes (283). Nevertheless, although all TRPMLs may have similar lysosomal functions, there exist some significant differences.

#### 4.2.3. TRPML2.

Unlike TRPML1, which is ubiquitously expressed, TRPML2 is more restrictively expressed, e.g., in the immune cells (278). Hypotonic challenge and physically pressing the endolysosomal membranes can potentiate the agonist-evoked TRPML2 currents but not agonist-evoked TRPML1 and TRPML3 currents (36, 284). Thus, TRPML2-mediated lysosomal Ca^2+^ release may play an important role in the osmoregulation of membrane trafficking (36, 284). Unlike the pan-TRPML agonist ML-SAs, which showed activation effects on all three TRPMLs (62, 285), a small-molecule ML2-SA1 (EVP-22) was recently identified as a relatively selective synthetic agonist of TRPML2 (61, 286). This selective agonist may help test whether TRPML2 regulates certain lysosomal functions independent of TRPML1. However, as discussed below, TRPML2 is also expressed in the recycling endosomes in addition to LELs.

#### 4.2.4. TRPML3.

TRPML3 is also restrictively expressed in some cell types, notably in cochlear hair cells, melanocytes, uroepithelial cells, and lung macrophages (221, 247, 274, 287–289). Unlike TRPML1 and TRPML2, which are potentiated by acidic luminal pH, TRPML3 is inhibited by acidic luminal pH (221, 275). This unique pH sensitivity may allow TRPML3 to regulate specific endolysosomal functions. For example, in bladder epithelial cells TRPML3 is disinhibited by lysosomal alkalization induced by pathogen invasion to mediate exosome release and bacterial extrusion (247). ML3-SA1 (EVP-77) was recently identified as a selective agonist for TRPML3 (61, 290). With the use of this selective agonist, it was shown that TRPML3 regulates early endosomal trafficking in alveolar macrophages (61, 290). However, given that TRPML3 is expressed in both LELs and early endocytic organelles, some of the observed functions of TRPML3 might be due to its expression in early endosomes and autophagosomes (discussed below).

#### 4.2.5. TPC1.

**FIGURE 7.**
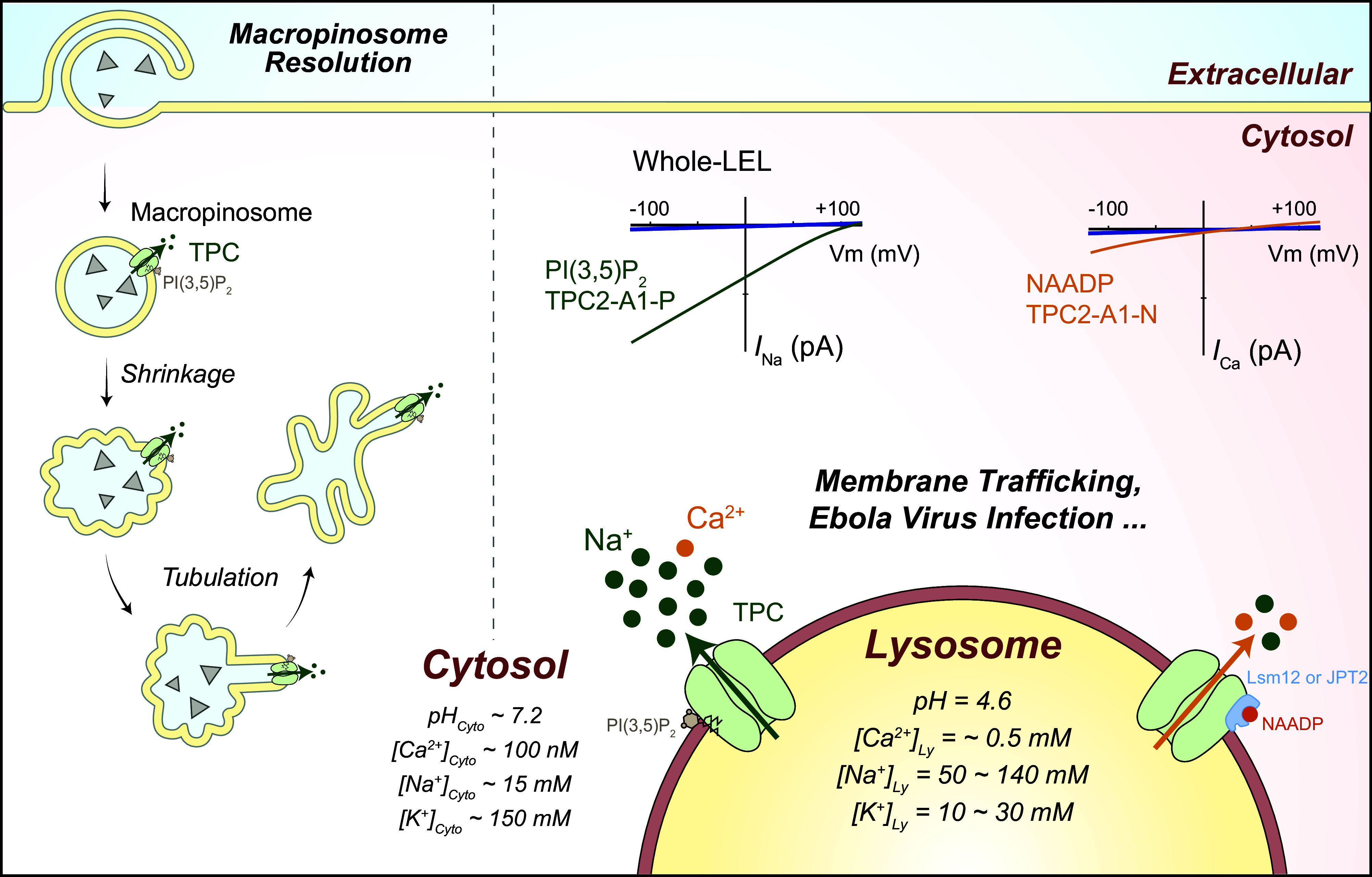
The channel and cell biological functions of two-pore channels (TPCs). The lysosomal lumen is a high-Na^+^ and -Ca^2+^ compartment. TPCs are highly Na^+^-selective channels with weak to medium Ca^2+^ permeability depending on the agonist type. Phosphatidylinositol 3,5-bisphosphate [PI(3,5)P_2_] and the synthetic agonist TPC2-A1-P preferentially activate TPCs’ Na^+^ conductance, although measurable Ca^2+^ influx can be detected in cells upon TPC2-A1-P application. TPC-mediated efflux of Na^+^, the major osmolyte in phagosomes and possibly in lysosomes, may release membrane tension to facilitate phagosome resolution and lysosome reformation. The activity of TPCs can be modulated by the ATP/mammalian target of rapamycin (mTOR) pathway. On the other hand, activation of TPCs by NAADP and its synthetic mimic TPC2-A1-N results in a significant increase in TPCs’ Ca^2+^ permeability. TPC-mediated lysosomal Na^+^ and Ca^2+^ release may together regulate membrane trafficking, exocytosis, lipid metabolism, and *Ebola* viral infection. LEL, late endosomes and lysosomes. *I*, current; *V*_m_, membrane potential.

Two-pore channel (TPC)1 and TPC2 are ubiquitously expressed LEL-localized cation channels in mammals. TPC1 is also localized in early endosomes and macropinosomes (50, 291). Each subunit of TPCs has two similar 6-TM helical repeats (IS1–IS6 and IIS1–IIS6), and two subunits are arranged in pseudotandem (291). Like canonical voltage-gated Na_V_/Ca_V_ channels, TPCs consist of one voltage sensor domain (S1–S4) that is connected to the pore domain formed by S5 and S6 (231). Whole LEL recordings revealed that TPC1 is a voltage-modulated PI(3,5)P_2_-activated Na^+^-selective channel with *P*_Na_/*P*_K_ of ∼70 and *P*_Na_/*P*_Ca_ of ∼10 (51, 292) (see FIGURE 7). Despite the presence of multiple positively charged residues in both IS4 and IIS4, only IIS4 was found to be responsible for TPC1’s voltage sensitivity (99, 292). The PI(3,5)P_2_-binding site is formed by the negatively charged amino acid residues in multiple domains, including IS3, IS4, IS6, and the IS4–S5 linker (292). In cells, TPCs are also found to be NAADP-sensitive lysosomal Ca^2+^ release channels (291, 293, 294) (see FIGURE 7). NAADP-binding sites are now known to be on accessory proteins such as LSM12 and JPT2 (295–299). However, whether NAADP may directly activate whole endolysosomal TPC1 currents still remains controversial (15, 51, 291, 292, 300–303); this issue is further discussed below. In addition, sphingosine was also reported to trigger TPC1-dependent Ca^2+^ release (304), but direct activation cannot be seen in the lysosomal patch-clamp studies (300). Hence, for studying NAADP and TPC regulation there may be discrepancies in the sufficiency versus necessity tests and in the channel-based versus cell-based assays.

PI(3,5)P_2_-activated whole LEL TPC1 currents can also be regulated by various cellular cues (302). Low luminal pH causes a dramatic rightward shift of the conductance-voltage (*G-V*) curve of TPC1 currents, suggesting that TPC1, like TRPML3, may not be maximally activated in the highly acidic LELs (99, 292). Whole LEL TPC1 current is inhibited by ATP/mTOR, suggesting that the metabolic state of the cell may regulate lysosomal Na^+^ flux (15).

TPC1 was found to be involved in macropinosome shrinkage in macrophages by promoting Na^+^ release (50). A similar mechanism might also be responsible for TPC1’s role in lysosomal reformation and tubulation (36). As lysosomal *E*_Na_ is greater than +50 mV, activation of TPCs should cause rapid lysosomal depolarization; the voltage dependence of TPC1 is implicated in the membrane excitability (rapid changes of Δψ) of lysosomes (99). TPCs were proposed to regulate lysosomal pH (15), but such results have not been confirmed by others (285, 302, 305). On the other hand, TPC-dependent Na^+^ or Ca^2+^ release was proposed to be responsible for endosomal trafficking of proteins, e.g., bacterial toxins, and various lysosomal functions, which include lysosomal trafficking, exocytosis, and organellar membrane contact (302, 305–309). To segregate the effects of various regulatory mechanisms, it is necessary to perform knockin mutational studies on the acting sites of PI(3,5)P_2_, NAADP, and mTOR.

#### 4.2.6. TPC2.

TPC2 is localized mainly on LELs (291, 294). The NH_2_-terminal dileucine lysosomal-targeting motif of TPC2 is responsible for its subcellular localization (51, 310). Like TPC1, TPC2 is also a PI(3,5)P_2_-activated Na^+^-selective channel (51, 292), but significant Ca^2+^ permeability is observed when TPC2 is activated by NAADP (306, 311). TPC2’s PI(3,5)P_2_-binding site is similar to that of TPC1 (312). Unlike TPC1, although there are several positively charged residues on both IS4 and IIS4, TPC2 currents lack apparent voltage sensitivity (292, 312).

Small-molecule synthetic compounds targeting TPCs have been identified (300, 306, 311). Several tricyclic antidepressants (TCAs) activate TPC2 in a voltage-dependent manner (300). In contrast, riluzole activates TPC2 independent of voltage, suggesting the existence of agonist-specific gating mechanisms (300) (TABLE 1). Two additional synthetic agonists have been identified for TPC2: TPC2-A1-N and TPC2-A1-P (311) (FIGURE 7 and TABLE 1). Intriguingly, whereas TPC2-A1-P preferentially activates the Na^+^ conductance, mimicking the effect of PI(3,5)P_2_, TPC2-A1-N renders the channel more Ca^2+^ permeable, resembling the effect of NAADP (306, 311). TPC’s relative Ca^2+^ permeability (*P*_Ca_/*P*_Na_) is increased from ∼0.1 to ∼0.7 if the channels are activated by NAADP and TPC2-A1-N (311). Hence, the pore properties of TPCs, i.e., the voltage dependence and cation selectivity, might differ depending on the activating cues of the channel. Nevertheless, activation of TPC2 by compounds that render high Na^+^ selectivity, such as TPC2-A1-P and riluzole, can also mediate Ca^2+^ flux in cells (51, 291, 300, 302, 311). Verapamil, Ned19, tetrandrine, as well as its derivatives such as SG-005 and SG-094, were reported to inhibit PI(3,5)P_2_-evoked TPC Na^+^ currents and TPC2-dependent cellular functions (51, 313, 314) (see TABLE 1).

Because TPC1 and TPC2 are either Na^+^ selective or Ca^2+^ permeable depending on the activation mechanism, the cell biological functions of TPCs can be grouped into Na^+^-dependent versus Ca^2+^-dependent functions. The Na^+^ influx through TPCs may release the primary osmolyte in the lumen, Na^+^, reducing luminal osmolarity to cause micropinosomal shrinkage (50). Similar mechanisms might also be required for lysosomal content condensation and hence reformation upon completion of degradation of endocytic, autophagic, and phagocytic cargo (20, 36). The activity of TPCs is regulated by the ATP/mTOR pathway, suggesting that lysosomal Δψ may change according to the metabolic status of the cells (15). It is worth noting that the Na^+^ conductance of TPCs may change lysosomal Δψ (lysosomal *E*_Na_ greater than +50 mV), which could indirectly affect Ca^2+^ release and Ca^2+^-dependent lysosomal functions (46). On the other hand, NAADP/TPC2-dependent local Ca^2+^ release may also subsequently trigger a global ER Ca^2+^ release via Ca^2+^-sensitive IP3Rs/RyRs through Ca^2+^-induced Ca^2+^ release (CICR) (306, 315) (FIGURE 4). As PI(3,5)P_2_/TPC2-A1-P may have synergistic effects with NAADP/TPC2-A1-N to promote Ca^2+^ flux through TPC2 (306), TPC2-A1-P could also play a role in lysosome exocytosis (311), which is thought to be a Ca^2+^-dependent process (316). Overall, TPCs are shown to be involved in diverse physiological functions, including autophagy (317), MCS formation (315), angiogenesis (318), muscle cell differentiation and contraction (319, 320), lipid metabolism (305), pathogen (e.g., Ebola virus) infection (309, 314), and starvation endurance (15) (FIGURE 7 and TABLE 1). In addition, aberrant regulation of TPC activity is also implicated in various pathologies including PD (321). Finally, although TPC1 and TPC2 share many lysosomal functions, there exist several isoform-specific roles of TPCs (99, 322).

#### 4.2.7. P2X4.

P2X4, as a member of the P2X receptor family of the ionotropic ATP receptor, is a Ca^2+^-permeable nonselective cation channel on the plasma membrane (323). However, P2X4, but not other P2X channels, is also expressed in LELs in some cell types (324). Hence, P2X4 can be a noncommitted organellar channel. By direct recording of membrane currents from enlarged LELs isolated from COS7 and HEK293 cells, lysosomal P2X4 was shown to be activated by ATP from the luminal side (see FIGURE 3). Activation of P2X4-dependent Ca^2+^ release was shown to promote lysosomal fusion in a CaM-dependent manner. Its activity is inhibited by an acidic luminal pH, similar to TRPML3 and TPC1 (324). Hence, luminal ATP increase and/or alkalinization may promote lysosomal fusion via P2X4. ATP intake into the lysosomes through SLC17A9, a solute carrier protein responsible for transporting ATP into lysosomes, readily activated lysosomal P2X4. By using P2X4 as a readout for luminal ATP, it was shown that lysosomal Δψ is the major driving force for SLC17A9-mediated lysosomal ATP transport (325). Hence, lysosomal ion channels and transporters that are involved in Δψ regulation and lysosomal acidification may regulate membrane trafficking indirectly through lysosomal P2X4.

#### 4.2.8. Lyso-BK/Lyso-K_VCa_.

The BK (big-conductance calcium-activated K^+^) channel, encoded by a single gene (*KCNMA1, SLO-1*) in mammals, belongs to the voltage-gated K^+^ channel (K_V_) superfamily (326). It is mainly expressed at the cell surface of the excitable cells, but low levels of expression can be detected in most cell types (45). BK channels can be dually activated by membrane depolarization and elevation of [Ca^2+^]_Cytosol_ and have a large single-channel conductance of 100–300 pS (326). Both endogenous and heterologously overexpressed BK channels are shown to be localized on the limiting membranes of LELs (45, 46) (see FIGURE 3). The LEL localization of BK is diminished by mutations in two dileucine motifs located in the cytosolic COOH terminus of BK (46). Outward-rectifying, voltage-dependent whole LEL BK-like currents were recorded in response to a cytosolic Ca^2+^ increase and membrane depolarization (Lyso-BK/Lyso-K_VCa_) in a variety of cell types (45, 46, 49, 239). Lyso-BK/Lyso-K_VCa_ currents were abolished in *Kcnma1* KO mouse fibroblasts but dramatically increased upon *KCNMA1*/*SLO1* overexpression (45, 46). Consistent with the plasma membrane BK currents, Lyso-BK currents were inhibited by known BK blockers, e.g., paxilline and iberiotoxin, but potentiated by BK channel openers, including NS1619 (326) (TABLE 1).

Lyso-BK channels regulate lysosomal functions through multiple mechanisms. First, an increase in juxtalysosomal Ca^2+^, e.g., through TRPML1 or TRPML3, may quickly activate Lyso-BK channels to cause lysosomal hyperpolarization (increasing lysosomal Δψ) (46, 327). As lysosomal *E*_K_ is more negative than lysosomal Δψ, activation of Lyso-BK may, in a positive-feedback mechanism, increase the driving force for further Ca^2+^ release through TRPML1/3 (46). Another feedback mechanism is that TRPML3 activity could be enhanced upon Lyso-BK activation to elevate [K^+^]_Lumen_ (327). Second, Lyso-BK is required for refilling lysosomal Ca^2+^ stores (45). Whereas upregulation of Lyso-BK facilitates lysosomal Ca^2+^ refilling, pharmacologically inhibiting Lyso-BK or mutating the Ca^2+^-binding site of BK suppresses lysosomal Ca^2+^ refilling (45). Hence, Lyso-BK is required for both release and uptake of lysosomal Ca^2 +^. Since lysosomal Ca^2+^ release may regulate various steps of lysosomal membrane trafficking, as discussed above, Lyso-BK may also regulate these processes. Consistently, knockout of Lyso-BK in human skin fibroblasts or mouse embryonic fibroblasts (MEFs) causes lysosomal enlargement and dysfunction (46, 239). Additionally, genetic deletion or pharmacological inhibition of Lyso-BK reduces proteolytic activity during starvation (45). Conversely, increasing Lyso-BK expression improves lysosomal membrane trafficking and mitigates the cellular phenotypes of various LSDs by facilitating lysosomal membrane trafficking (239). Hence, Lyso-BK may regulate lysosomal functions via lysosomal Δψ and Ca^2+^ signaling.

#### 4.2.9. Lyso-volume-regulated anion channel.

**FIGURE 8.**
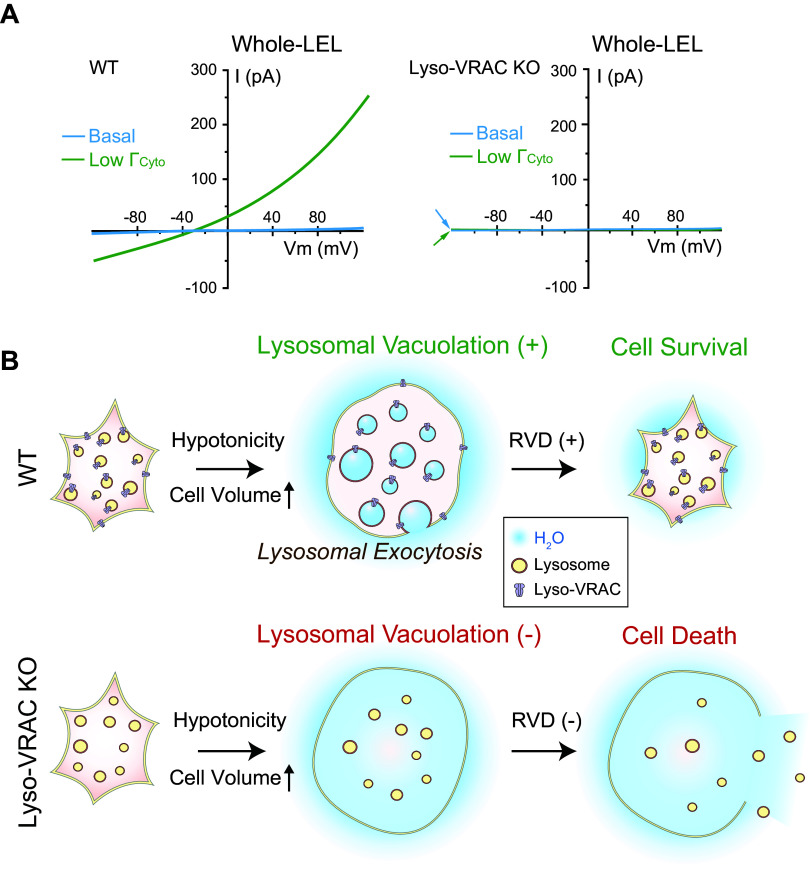
Lyso-VRAC in lysosome volume and cell volume regulation. *A*: low ionic strength (Γ)-activated whole late endosome and lysosome (LEL) Cl^−^ currents (Lyso-VRAC) in wild-type (WT) and LRRC8A knockout (KO) cells. *I*, current; *V*_m_, membrane potential. *B*: a working model illustrating the roles of lysosomes and Lyso-VRAC in cellular osmoregulation upon hypoosmotic stress. Hypotonic challenges result in water influx to decrease the osmolarity of the cytosol, and the reduced cytosolic osmolarity and ionic strength activate Lyso-VRAC. Lysosomal vacuolation occurs in a Lyso-VRAC-dependent manner to create large-volume intracellular compartments for excess water storage. Lysosomal water uptake and subsequent vacuolation restore the cytosolic volume, which is required for normal cellular metabolism. Exocytosis of water-filled lysosomal vacuoles, i.e., lysosomal exocytosis, not only extrudes excess water to the extracellular space but also reduces plasma membrane tension stress by delivering intracellular membranes to the plasma membrane (cell survival, *top*). In the cells that lack Lyso-VRAC, lysosome vacuolation is blocked, resulting in necrotic cell death due to an impairment in regulatory cytosolic volume decrease (cytosolic RVD; cell death, *bottom*).

Volume-regulated anion channels (VRACs) play a key role in regulated cell volume decrease (RVD), as an adaptive response to hypotonic stress, by mediating the efflux of Cl^−^ and organic osmolytes followed by the osmotically obligated water flux (328, 329). VRACs are composed of LRRC8 family proteins; the essential subunit LRRC8A/SWELL1 forms a hexameric complex with other subunits, including LRRC8B–E (328, 329). VRACs are activated by reduced cytosolic osmolality or ionic strength (328, 330, 331) and are permeable to Cl^−^ as well as some organic solutes such as glutamate, taurine, and ATP (330) (FIGURE 8 and TABLE 1). LRRC8A is ubiquitously expressed in many tissues and can traffic to LELs via a lysosome-targeting motif to form Lyso-VRAC, mediating Cl^−^ flux across lysosomal membranes (115, 330) (FIGURE 8). [Cl^−^]_Lysosome_ is estimated to be ∼80–100 mM, which is several-fold higher than cytosolic [Cl^−^] (FIGURE 3), and hence lysosomal *E*_Cl_ is approximately −30 mV (cytosolic side negative) under resting conditions (52, 53). Therefore, with resting Δψ of −25 mV, the direction of lysosomal Cl^−^ flux through Lyso-VRAC may be dependent on lysosomal Δψ changes. Like plasmalemmal VRACs, Lyso-VRACs are activated by cytosolic hypotonicity or low ionic strength (FIGURE 8) and are permeable to Cl^−^, as well as other anions such as glutamate and $\mathrm{HC}O_{3}^{-}$ (115). Under hypotonic stress, Lyso-VRACs are required for RVD through two different mechanisms. First, Lyso-VRACs mediate osmolyte flux, followed by osmotically coupled water flux into the lysosome lumen (lysosomal vacuolation), effectively reducing the water concentration in the cytosol and hence cytosolic RVD (36, 115) (FIGURE 8). Second, via a mechanism that is not yet clear, Lyso-VRACs are required for the exocytosis of water-filled vacuolated lysosomes and effectively reducing the water concentrations in the cytoplasm (36) (FIGURE 8). Similar active water extrusion mechanisms have been described for contractile vacuoles in unicellular protists, such as social amoeba (36, 332). Finally, Lyso-VRACs may also regulate certain luminal functions through lysosomal Cl^−^ flux, as intralysosomal Cl^−^ accumulation is shown to be required for hydrolase activation (54, 55).

#### 4.2.10. TMEM175.

TMEM175 is a ubiquitously expressed LEL-localized cation channel, but small amounts of TMEM175 puncta can also be detected in early endosomes (104, 333). Structural studies revealed that human TMEM175 forms a dimeric 12-TM channel (334–337). TMEM175-specific currents have been successfully recorded by whole LEL patch clamp (104, 338) (FIGURE 2). Recent genome-wide association studies (GWAS) have identified TMEM175 variants as a risk factor for PD (339–342) and related α-synucleinopathies (104, 205, 343, 344).

##### 4.2.10.1. channel permeation and selectivity.

**FIGURE 9.**
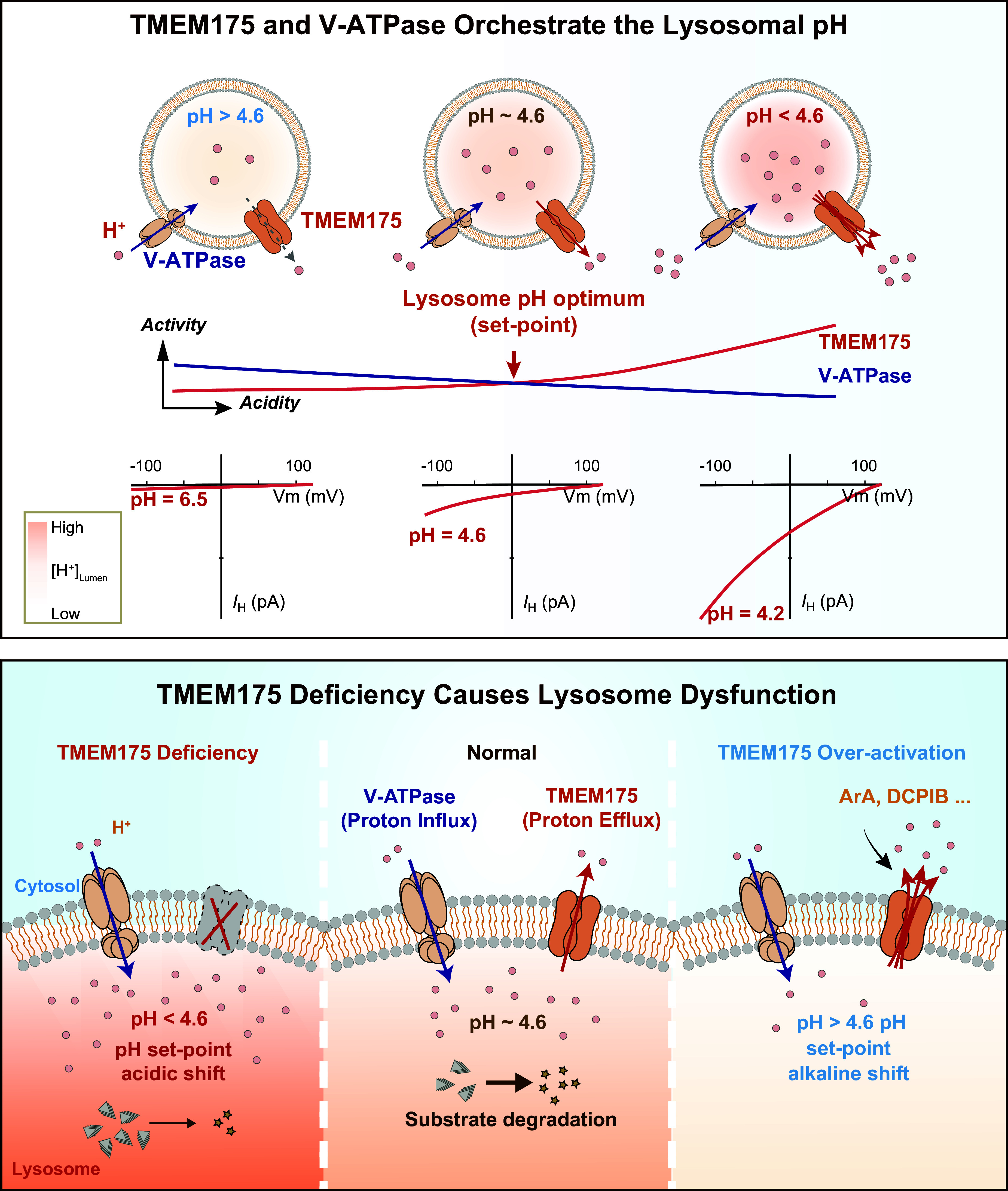
TMEM175 in lysosomal pH regulation. Lysosomal pH is heterogeneous within the range of 4.5–5.0 that is required for the optimal hydrolytic activity of most lysosomal enzymes. For individual lysosomes, the steady-state luminal pH is determined by the relative rates of vacuolar-type (V-)ATPase-mediated proton influx (which decreases with luminal acidification) vs. TMEM175-mediated proton efflux (which increases dramatically with luminal acidification). For a typical lysosome with a steady-state pH of 4.6 (the set point), a small TMEM175 H^+^ current is needed to balance the V-ATPase-mediated proton influx. Acidification below the set point dramatically activates TMEM175 currents to prevent further acidification via a negative-feedback regulation mechanism. Increasing the expression/activity of TMEM175 causes a transient alkaline shift in the steady-state set-point pH. TMEM175 deficiency causes lysosomal overacidification and reduced activities of lysosomal hydrolases. ArA, arachidonic acid; DCPIB, 4-[(2-butyl-6,7-dichloro-2-cyclopentyl-2,3-dihydro-1-oxo-1*H*-inden-5-yl)oxy]butanoic acid; *I*, current; *V*_m_, membrane potential.

TMEM175 was initially reported to mediate a constitutively active background K^+^ conductance of the early endosomes and lysosomes (333). However, in most cell types TMEM175-dependent basal lysosomal K^+^ currents are very small, and nondetectable in most isolated lysosomes (45, 46, 104), suggesting that TMEM175 might be further potentiated by certain cellular cues under physiological conditions. One potential candidate is luminal pH, as TMEM175 is proposed to maintain lysosomal pH homeostasis during starvation (333). Several recent studies revealed that TMEM175 is substantially more permeable to H^+^ compared with Na^+^ or K^+^ (104, 105, 338). TMEM175’s K^+^ conductance is suppressed at lysosomal acidic pH, and the relative permeability of H^+^ over K^+^ and other ions, including Na^+^ and NMDG^+^, is up to 50,000 fold (104, 105). Hu et al. showed that TMEM175 may function as a H^+^-activated H^+^-selective channel in the LELs, acting as a balancer for the V-ATPase to maintain lysosomal pH optimum (103–105, 123) (FIGURE 9). Each TMEM175 subunit harbors two FSD motifs on TM1 and TM7, which are likely responsible for a high H^+^ over K^+^/Na^+^ selectivity (105, 334). In the cryo-EM structures, it was shown that the pore radius is smaller under acidic pH conditions compared with neutral pH conditions, and such a mechanism may be what arrests the movement of K^+^ ions through TMEM175 (105). Consistent with the electrophysiological recordings, organellar pH imaging studies (FIGURE 2*B*) showed that activation of TMEM175 decreases juxtalysosomal pH but increases luminal pH (104). Whereas the V-ATPase is responsible for H^+^ pumping into the lumen of lysosomes, TMEM175 can prevent lysosomal overacidification, such that lysosomal hyperacidification beyond the normal range (i.e., <pH 4.5) can dramatically activate TMEM175 to facilitate lysosomal H^+^ release (103, 104) (see FIGURE 9). It remains untested whether TMEM175-mediated K^+^ flux may play a physiological role in less acidified lysosomes or early endosomes. Several pore mutations in TMEM175 were found to selectively reduce or abolish the H^+^ conductance without affecting the K^+^/Cs^+^ conductance (104, 105). Hu et al. (103, 104) found that one of these H^+^ conductance-selectively deficient mutations, D41A, failed to rescue the lysosomal hyperacidification phenotype in TMEM175 KO cells. However, it is not clear whether D41 is required for H^+^ activation or H^+^ permeation.

##### 4.2.10.2. channel modulation.

###### 4.2.10.2.1. Luminal protons.

TMEM175-dependent macroscopic H^+^ currents are dramatically increased when the luminal pH is decreased (103, 104). In a carefully designed experiment, it was demonstrated that luminal protons, but not cytosolic protons, indeed have an activation gating effect (104). In other words, luminal H^+^ may act as an endogenous activator to open the TMEM175 channels (104, 105). A plasmalemmal H^+^ channel that belongs to the OTOP family was also recently shown to be H^+^ gated (345). Hence, TMEM175 may function as a lysosomal proton-activated proton channel (LyPAP). As a LyPAP, TMEM175 regulates the lysosomal pH set point via a classic negative-feedback loop mechanism (FIGURE 9), such that TMEM175 integrates both the sensor and the response element within one protein (103). In the native lysosomes, TMEM175 is likely to be only minimally active, but lowering luminal side pH to below pH 4.6 dramatically increases the H^+^ channel activity (104) (FIGURE 9). In the endogenous setting, the lysosome pH set point may serve as the “threshold” pH for TMEM175 activation (103, 104). Hence, TMEM175 may regulate lysosomal pH homeostasis as a H^+^ overload-activated H^+^ channel, analogous to how TMCO1 regulates ER Ca^2+^ homeostasis as a Ca^2+^ overload-activated Ca^2+^ channel.

###### 4.2.10.2.2. Arachidonic acid.

A polyunsaturated lipid, arachidonic acid (ArA), can activate whole LEL TMEM175 H^+^ currents (TABLE 1), suggesting that ArA may serve as an endogenous cellular cue to regulate endosomal and lysosomal pH (104). Consistently, ArA is known to induce lysosomal membrane permeability to H^+^ and K^+^ in isolated lysosomes (346). However, TMEM175 is barely activated by early endosomal pH ∼5.5, suggesting that in early endosomes ArA may shift TMEM175’s pH sensitivity toward the less acidic range (104). Consistently, ArA is reported to enhance endosomal fusion (347, 348), and inhibition of phospholipase A_2_ (PLA_2_), which is mainly responsible for cytosolic ArA elevation, blocks endosome fusion events (348). However, the concentration of ArA (tens of micromolar) that is needed to activate TMEM175 is rather high. To establish a physiological role of ArA in TMEM175-mediated endosomal and lysosomal functions, it is necessary to structurally reveal the ArA-binding sites and conduct ArA-insensitive knockin mutational studies.

###### 4.2.10.2.3. AKT and Bcl-2.

AKT (also known as protein kinase B or PKB) is a serine/threonine kinase that plays a key role in regulating various cellular processes such as cell survival and growth (349). A recent study reported that SC-79, a small-molecule activator of AKT, increased the whole LEL K^+^ currents in TMEM175-transfected HEK293 cells via a catalysis-independent but interaction-dependent mechanism (343). However, Hu et al. (104) failed to observe any significant activation effect of SC-79 on either TMEM175 H^+^ or K^+^ current. Bcl-2 (B cell lymphoma 2), a protein that is known to regulate apoptosis, was reported to interact directly with TMEM175 to regulate its channel activities (350). However, the Bcl-2-dependent effect on TMEM175 currents was quite slow and small compared with the fast and robust activation effects of H^+^ and ArA, so its physiological significance is not clear.

###### 4.2.10.2.4. Synthetic agonists and inhibitors.

Several synthetic compounds were found to modulate TMEM175’s channel activity. 4-[(2-Butyl-6,7-dichloro-2-cyclopentyl-2,3-dihydro-1-oxo-1*H*-inden-5-yl)oxy]butanoic acid (DCPIB), a widely used VRAC blocker, was found to be an activator of TMEM175’s H^+^ and K^+^ currents in both overexpression and endogenous systems (104) (TABLE 1). DCPIB was also shown to induce TMEM175-dependent lysosomal H^+^ release (104). Likewise, ML 67-33 was also identified as a synthetic agonist of TMEM175 (TABLE 1). On the other hand, 4-aminopyridine (4-AP) was found to be a low-affinity inhibitor of TMEM175 (351) (TABLE 1). Future development of highly potent TMEM175-specific small-molecule modulators may help define the direct cell biological roles of TMEM175.

##### 4.2.10.3. cell biological functions.

###### 4.2.10.3.1. Lysosomal pH regulation.

Lysosomes establish and maintain a >500-fold [H^+^] gradient across their membranes through V-ATPase (29) (FIGURE 3). Because pharmacological inhibition of the V-ATPase by Bafilomycin-A1 leads to rapid lysosomal deacidification, there must exist an unidentified “proton leak” conductance (LysoH) on lysosomal membranes (25, 81, 102–104). The proton leak current is absent in TMEM175 KO cells but dramatically increased upon TMEM175 overexpression, so TMEM175 is likely the molecular basis of LysoH (104, 105). Moreover, agonist-induced activation of TMEM175 results in an increase in the luminal pH with a simultaneous decrease in the juxtalysosomal pH (104). Hence, acting as a proton-activated proton channel (LyPAP), TMEM175 regulates the lysosomal pH set point and optimum to prevent lysosomal overacidification (104, 105) (see FIGURE 9).

Most lysosomal hydrolases require an acidic lumen for their optimal activities (209). For instance, Cathepsin B, a cysteine protease in the lysosome, reaches its peak degradative activity at pH ∼5 (352). Loss of TMEM175 impairs the overall degradation capability of lysosomes, as assayed by DQ-BSA (104). The activities of Cathepsin B and Cathepsin D are both compromised in TMEM175 KO cells (104, 343, 344). Lysosomal H^+^ flux may also regulate other lysosomal functions. For example, a lysosomal H^+^ gradient is required for driving solute flux through H^+^-coupled ion and catabolite transporters (196, 353, 354). In addition, lysosomal H^+^ release is proposed to regulate various lysosomal trafficking steps, which include lysosomal mobility as well as endosomal sorting complex required for transport (ESCRT)-dependent inward budding and formation of intraluminal vesicles (7, 13). Furthermore, luminal H^+^ also regulates the activities of various lysosomal ion channels, including TRPML1, TRPML3, TPC1, and P2X4 (99, 221, 355). As TMEM175 is dually permeable to H^+^ and K^+^, it remains to be determined whether TMEM175’s proton conductance plays a role in regulating these lysosomal functions.

Many lysosome-related diseases, including PD, AD, and LSDs, exhibit abnormal lysosomal acidification (213, 356–358); normalizing lysosomal pH pharmacologically has been shown to restore lysosomal functions in these disease pathologies (213, 359, 360). Hence, targeting TMEM175 with small-molecule modulators could provide a therapeutic approach to treating various lysosomal diseases that are caused by defects in lysosomal acidification.

###### 4.2.10.3.2. Lysosomal Δψ regulation.

Because lysosomal *E*_H_ (greater than +150 mV) is much more positive than the resting lysosomal Δψ (106) (FIGURE 3), TMEM175-dependent lysosomal H^+^ efflux may contribute significantly to lysosomal depolarization. Consistently, proton ionophores are known to cause lysosomal depolarization (45). Hence, TMEM175 may mediate nutrient- and pH-dependent regulation of lysosomal Δψ.

###### 4.2.10.3.3. Autophagosome-lysosome fusion.

Lysosomes are required for the degradation of autophagic cargos. Besides its role in lysosomal degradation, lysosomal pH may play an important role in autophagosome-lysosome fusion (28, 361) (FIGURE 1). With the use of the GFP-red fluorescent protein (RFP)-LC3 reporter to monitor autophagic flux, autophagosome-lysosome fusion was found to be accelerated in TMEM175 KO cells (333). It remains to be determined whether the observed phenotype is caused by lysosomal overacidification (104). Mitochondrial dysfunction is known to be associated with PD pathology (298). TMEM175 KO cells exhibit mitochondrial dysfunction and oxidative stress (333, 344, 350). It remains to be determined whether lysosomal overacidification caused by TMEM175 deficiency is responsible for mitochondrial dysfunction and PD pathology.

#### 4.2.11. CLN7.

CLN7 (MSFD8) belongs to the major facilitator superfamily (MFS) with 12 predicted transmembrane domains (110, 362). CLN7 is primarily localized on LELs with its NH_2_ and COOH termini facing the cytosol (362). An NH_2_-terminal dileucine-based motif is required for the LEL localization of CLN7 (362). Mutations of CLN7 underlie a form of LSD called variant late-infantile neuronal ceroid lipofuscinosis (vLINCL) (363). CLN7 was recently shown to be an endolysosomal Cl^−^ channel (110). Whereas overexpression of CLN7 was found to increase lysosomal Cl^−^ currents that are sensitive to known Cl^−^ channel blockers (TABLE 1), basal lysosomal Cl^−^ currents were reduced in the CLN7 KO cells but modestly increased upon CLN7 expression (110). Overexpression of an NH_2_-terminally tagged construct of CLN7 caused an enlargement of lysosomes in a Ca^2+^/calmodulin-dependent manner (110). It was shown that manipulating the expression and activities of CLN7 caused changes in the lysosomal Cl^−^ content, luminal pH, and lysosomal Δψ, as well as TRPML1-mediated lysosomal Ca^2+^ release (110). At the organismal level, genetic inactivation of CLN7 in mice caused characteristic LSD-like phenotypes resembling those observed in vLINCL patients, including retinal degeneration and autofluorescent lipofuscin accumulation (110).

#### 4.2.12. Other lysosomal channels.

Several plasma membrane channels were also found to be localized in the lysosomes of certain cell types and may function as noncommitted organellar channels. For example, TRPM2 was found to be localized in the LAMP1-positive compartments of pancreatic β-cells (364). Additionally, TRPA1 might mediate agonist-induced lysosomal Ca^2+^ release in dorsal root ganglion (DRG) neurons to regulate neuropeptide release (365). Furthermore, ectopic overexpression of TWIK2 increased basal lysosomal K^+^ currents and altered lysosomal functions (366). Hence, in certain cell types expressing endogenous TWIK2 lysosomal Δψ is likely different compared with cells lacking TWIK2 (49). In *Drosophila* and mouse neurons, a Ca_V_ channel was found to be localized in lysosomes, potentially regulating Ca^2+^-dependent autophagosome-lysosome fusion (367). It remains to be determined whether Ca_V_, Na_V_, or K_V_ channels are functionally expressed in the LELs of mammalian neurons. Finally, some lysosomal membrane proteins, e.g., SIDT2, are reported to function as cation channels at the plasma membrane (368), but confirmations with lysosomal electrophysiology are warranted.

### 4.3. Ion Channels in the Early Endosomes, Recycling Endosomes, Macropinosomes, Phagosomes, and Autophagosomes

#### 4.3.1. Early endosomes.

**FIGURE 10.**
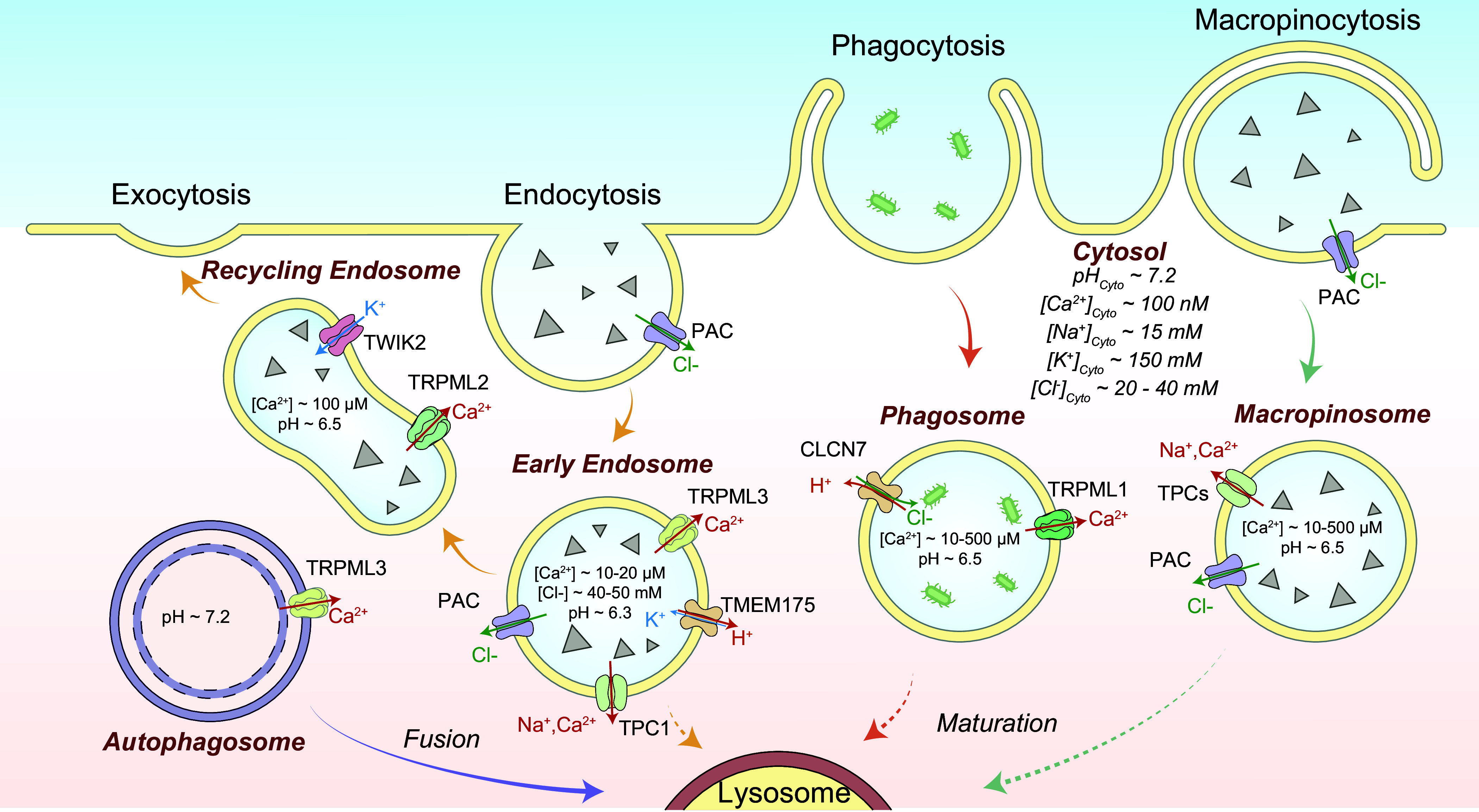
Ion channels in the autophagic and early endocytic pathways. Patch-clamp and immunofluorescence assays revealed the expression of several ion channels in the early endosomes, recycling endosomes, macropinosomes, and autophagosomes. Whereas TRPML2 is expressed in the recycling endosomes [in addition to late endosomes and lysosomes (LELs)], TRPML3 is expressed in both early endosomes and autophagosomes (in addition to LELs). Two-pore channels (TPCs) are Na^+^ (Ca^2+^) channels in the macropinosomes and phagosomes; proton-gated anion channel (PAC)/TMEM206 mediates a Cl^−^ conductance in early endosomes and macropinosomes. There are progressive changes in the ionic compositions of early endocytic organelles. For example, endosomes are quickly acidified by vacuolar-type (V-)ATPase-mediated H^+^ pumping into the lumen, and luminal Ca^2+^ concentration ([Ca^2+^]) is quickly reduced to tens of micromolar, compared with ∼2 mM in the extracellular space.

*TMEM206* was recently identified to encode a proton-gated anion channel (PAC) in the plasma membrane of many cell types (369, 370). TMEM206 is evolutionarily conserved and widely expressed in various human tissues (369, 370). Acidic extracellular pH (<5.5) elicited outward-rectifying whole cell Cl^−^ currents in WT but not TMEM206 KO cells (369, 370). PAC, a trimeric channel formed by three identical 2-TM subunits as revealed by cryo-EM structural analysis (296), is permeable to Cl^−^, Br^−^, $NO_{3}^{-}$, I^−^, and SCN^−^ (369). Several charged amino acid residuals located extracellularly are potential H^+^ sensors required for proton gating (371). Plasmalemmal PACs may translocate through the endocytic pathway to macropinosomes and early endosomes, but not lysosomes, resulting in whole early endosomal PAC currents (125, 372) (FIGURE 10). PAC deficiency causes a loss of early endosomal Cl^−^ currents as well as endosomal Cl^−^ accumulation, hyperacidification, and endocytosis block defects (125). Hence, PACs are functionally active in the early endocytic pathway, regulating endosomal H^+^ and Cl^−^ homeostasis (125).

TRPML3 is an endosomal channel in macrophages (32, 277, 287, 290) (FIGURE 10), and TRPML3 deficiency in these cells results in an impairment in endolysosomal trafficking and macropinocytosis (290). As acidic pH has been shown to inhibit TRPML3, TRPML3 may be more active in less acidic early endosomes compared with LELs (247, 275). On the other hand, phosphatidylinositol 3-phosphate [PI(3)P], a phosphoinositide enriched on the membranes of early endosomes, may directly activate endosomal TRPML3 to regulate membrane trafficking in the early endocytic pathway (32, 73) (see TABLE 1). Similarly, TPC1 may also be an early endosomal channel that is inhibited by acidic pH and activated by PI(3)P (309, 373) (see TABLE 1).

#### 4.3.2. Macropinosomes.

Macropinocytosis is a particular form of endocytosis that takes up large amounts of fluids into the cells. Shrinkage of macrophage macropinosomes, an essential process for macropinocytosis, is impaired in TPC and PAC KO cells (50, 372). It was proposed that TPC-mediated Na^+^ efflux and Cl^−^ efflux through PAC channels are required for content (osmolyte) condensation in macropinosomes (FIGURE 10), in which the cellular cues that activate TPCs and PACs are likely PI(3,5)P_2_ and luminal H^+^, respectively (50, 372).

#### 4.3.3. Phagosomes.

TRPML1 is a committed lysosomal channel that regulates lysosomal membrane fusion and fission (56). However, in macrophages undergoing phagocytosis of large particles, such as apoptotic cells, TRPML1 may be recruited to the nascent phagosomes via focal exocytosis of lysosomes (88). Whole phagosome recordings confirmed the functional expression of TRPML1 in the nascent phagosomes (88). Likewise, phagosome-localized CLCN7/ClC7, a lysosomal H^+^/Cl^−^ exchanger, may mediate Cl^−^ flux required for phagosome resolution and protection of phagolysosomal membrane integrity in vivo (54, 55). Moreover, phagolysosomal CLCN7 is responsible for the creation and maintenance of a trans-lysosomal Cl^−^ concentration gradient to support the activation of hydrolases (54, 55).

#### 4.3.4. Recycling endosomes.

In the immune cells, TRPML2 is functionally localized in recycling endosomes (25, 277, 278) (FIGURE 10). Whereas TRPML2 deficiency impairs the recruitment of macrophages upon LPS treatment and bacterial infection (278, 286), LPS stimulation led to more endolysosomal TRPML2 currents evoked by the selective agonist ML2-SA1 (61, 286) (TABLE 1). Endolysosomal TRPML2 is sensitive to osmomechanical stress, suggesting the roles of TRPML2 in osmo-induced endosomal Ca^2+^ release and subsequent Ca^2+^-dependent endosomal membrane trafficking (284). TRPML2-mediated endosomal Ca^2+^ release may affect cytokine release (286). However, the recycling endosomal functions of TRPML2 are not firmly established, as lysosomal defects associated with TRPML2 deficiency could indirectly affect the functions of early and recycling endosomes (25, 277). A recent study showed that in macrophages TWIK2 is a recycling endosome-localized K^+^ channel that regulates luminal [K^+^] (374).

#### 4.3.5. Autophagosomes.

Several key steps of autophagy are thought to be Ca^2+^ dependent (86, 206, 375). As the liquid contents of the autophagosomes are derived from the cytosol, the nascent autophagosomes likely contain high K^+^ but low Na^+^ or Ca^2+^, with *E_x_* ∼0 mV. Besides early endosomes and LELs, TRPML3 was also found to be localized on autophagosomes, in which PI(3)P, synthesized by the VPS34 complex, becomes enriched upon autophagy induction (73, 376). PI(3)P may directly activate autophagosome-localized TRPML3 to provide Ca^2+^ for phagophore formation and autophagic membrane fusion (73). However, the generation of PI(3)P may require TRPML1-mediated lysosomal Ca^2+^ release, which can help initiate phagophore formation through CaMKKβ-dependent formation of the VPS34 complex (204). Hence, LEL-localized TRPMLs and autophagosome-localized TRPML3 may regulate both the early and late stages of autophagy. For the autophagosomes to release Ca^2+^ through TRPML3, one must assume that there also exists a Ca^2+^ gradient across autophagosomal membranes. However, it is not clear whether and how autophagosomes acquire Ca^2+^.

### 4.4. Ion Channels in the Endosome- and Lysosome-Related Organelles: Melanosomes and Tubulovesicles

In the specialized cell types, there exist endosome- and lysosome-related organelles. For instance, synaptic vesicles and melanosomes are both endosome-related organelles in neurons and melanocytes, respectively. Several endolysosomal channels are reportedly present in these cell type-specific organelles.

#### 4.4.1. Melanosomes.

**FIGURE 11.**
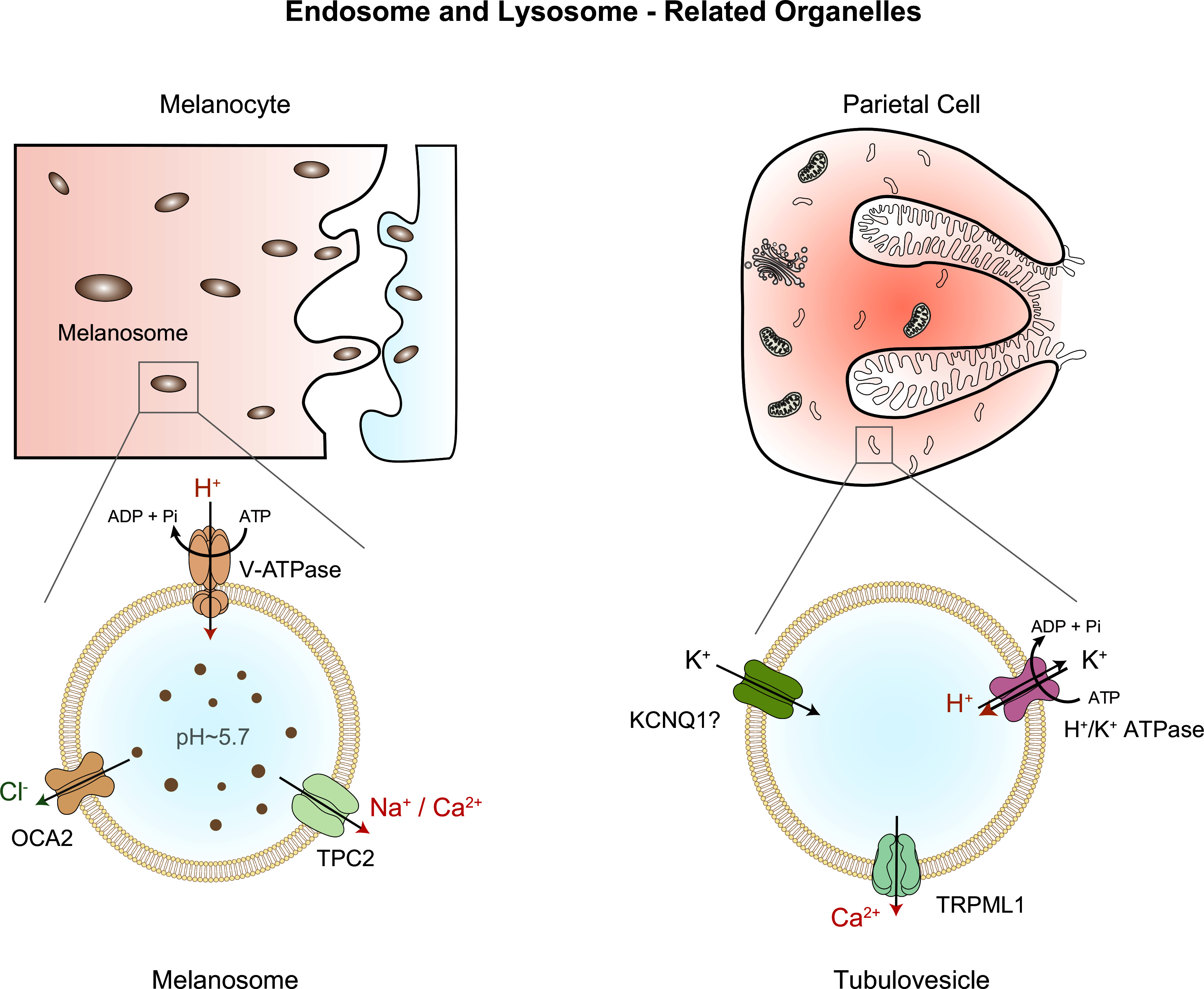
Ion channels in the endosome- and lysosome-related organelles. Endosome- and lysosome-related organelles are present in certain cell types, such as melanosomes (*left*) in melanocytes and tubulovesicles (TVs, *right*) in parietal cells. Whole melanosome recordings reveal the presence of TPC2 and OCA2 channels in melanosomes. The acidic pH gradient of melanosomes is produced by vacuolar-type (V-)ATPase pumping H^+^ to reach a luminal pH of ∼5.7. TPC2-mediated Na^+^/Ca^2+^ flux and OCA2-mediated Cl^−^ flux regulate melanosomal acidification and melanin production. *Right*: organellar patch clamp has been employed to record TRPML1 currents in tubulovesicular membranes. The H^+^-K^+^-ATPase on TVs translocates to the apical membrane (TV exocytosis) upon stimulation to pump H^+^ to the stomach lumen. TRPML1-mediated TV Ca^2+^ release promotes histamine-induced TV exocytosis and gastric acid secretion.

TPC2 is found to be functionally expressed in the melanosomes. TPC2 was shown to regulate melanosomal pH (321, 377–380), which is in turn required for the optimal activities of tyrosinases, melanogenesis, and melanosome maturation (381) (FIGURE 11). Gain-of-function mutations of TPC2, e.g., M484L, G734E, and R210C, cause hypopigmentation in humans (377, 379). OCA2, a 12-TM membrane protein, is also found to be localized on the melanosomes (FIGURE 11). Mutations in *OCA2* may underlie type 2 oculocutaneous albinism (TABLE 2). With support from direct patch-clamp recordings of enlarged melanosomes, OCA2 is reported to function as a melanosome-specific anion channel that regulates melanosomal pH and melanin production (381).

**Table 2.** Endomembrane channels in disease

Name	Organellar and Cellular Functions	Animal Phenotype	Human Disorder	Drug Target
*Committed organellar channels*				
*Secretory pathway*				
IP_3_Rs	ER/SR Ca^2+^ release	Ataxia, epilepsy, movement disorder, exocrine dysfunction, and distorted taste perception	Spinocerebellar ataxia, Gillespie syndrome, anhidrosis, CMT1J	
RyRs	ER/SR Ca^2+^ release	Embryonic cardiac lethality	Malignant hyperthermia, central core disease, CPVT, ventricular fibrillation, King–Denborough syndrome	
TRICs	Counterion K^+^ flux for ER/SR Ca^2+^ release	Embryonic cardiac lethality, osteogenesis imperfecta		
TMCO1	ER Ca^2+^ homeostasis	Delayed osteogenesis, craniofacial dysmorphism, mental retardation, and ataxia	CFSMR1 (OMIM#213980),	
CLCC1	Counterion Cl^−^ flux for ER/SR Ca^2+^ release	Sensitive to ER stress challenge	ALS	
*Degradative pathway*				
TRPML1	Lysosome Ca^2+^ signaling, membrane trafficking, lysosomal fusion, lysosomal fission, lysosomal exocytosis, lysosome movement, autophagy, MCS formation, and lysosome Fe^2+^/Zn^2+^ release	Neurodegeneration, retinal degeneration, hypochlorhydria, and muscular dystrophy	ML-IV, NPC	ML-IV, LSDs, PD, AD
TRPML2	Lysosome Ca^2+^ signaling, lysosomal osmosensitivity	Impaired innate immunity responses		
TRPML3	Lysosome Ca^2+^ signaling, bacterial extrusion	Varitint-waddler (GOF), emphysema		Chronic obstructive pulmonary diseases
TPC1	Macropinosome shrinkage, lysosomal Δψ regulation, pathogen invasion, and endosomal trafficking of bacterial toxins			
TPC2	Melanosomal acidification, lysosome Δψ regulation, macropinosome shrinkage, pathogen invasion, and cholesterol accumulation	Starvation endurance	Hypopigmentation (GOF), fatty liver disease	Ebola virus infection
TMEM175	Lysosomal acidification regulation, lysosome Δψ regulation, and membrane trafficking	Neurodegeneration	PD, sleeping disorder	PD
CLN7	Lysosomal acidification regulation	Retinal degeneration	Neuronal ceroid lipofuscinoses	
OCA2	Melanosome acidification	Hypopigmentation	Oculocutaneous albinism	

AD, Alzheimer’s disease; ALS, amyotrophic lateral sclerosis; CFSMR1, craniofacial dysmorphism, skeletal anomalies, and impaired intellectual development syndrome-1; CMT1J, Charcot-Marie-Tooth disease type 1 J; CPVT, catecholaminergic polymorphic ventricular tachycardia; ER, endoplasmic reticulum; GOF, gain of function; IP_3_, inositol (1,4,5)-trisphosphate; IP3R, IP_3_ receptor; LSD, lysosome storage disease; MCS, membrane contact site; ML-IV, mucolipidosis type IV; NPC, Niemann–Pick disease type C; PD, Parkinson’s disease; RyR, ryanodine receptor; SR, sarcoplasmic reticulum; TRIC, Trimeric intracellular cation channel; Δψ, membrane potential.

#### 4.4.2. Tubulovesicles.

In the specialized cell types, TRPMLs are also expressed in endosome- or lysosome-related vesicles such as tubulovesicles (TVs), which are specialized organelles of acid-secreting gastric parietal cells (382). TRPML1 is required for TV Ca^2+^ release that triggers TV exocytosis in response to cAMP/PKA signaling downstream of the receptor for histamine, a neurotransmitter that induces gastric acid secretion (134) (FIGURE 11). TV exocytosis is essential for the translocation of H^+^-K^+^-ATPase to the apical membranes of parietal cells in response to histamine (134, 382). Note that H^+^-K^+^-ATPase inhibitors have been used to treat hyperchlorhydria, suggestive of the druggability of organellar channels/transporters. TV-localized TRPML1 can be seen as a receptor-operated channel. Consistently, whereas genetic inactivation or pharmacological inhibition of TRPML1 suppresses gastric acid secretion, TRPML1 overexpression or activation augments gastric acid secretion in mice (134, 383). cAMP/PKA signaling reportedly regulates TRPML1 and some lysosomal functions (384, 385). In parietal cells, histamine-stimulated PKA phosphorylation increases the activity of TV-localized TRPML1 channels, resulting in increases of TV Ca^2+^ release and gastric acid secretion (134, 384) (see FIGURE 11).

## 5. ENDOMEMBRANE ION CHANNELS IN DISEASES

Mutations of several endomembrane channels are known to cause various human diseases by compromising the functions of their resident organelles (TABLE 2). In addition, some related human disorders are caused by defects in the signaling pathways that are upstream of the endomembrane channels or in their downstream effectors. Hence, small-molecule synthetic modulators (activators and inhibitors) of the endomembrane channels may provide a therapeutic approach to treat these diseases by restoring normal organelle function. Importantly, the identification and characterization of several endomembrane organellar channels have laid a solid foundation for developing channel-based HTS assays to screen for small-molecule modulators of endomembrane channels. Proof-of-concept studies have demonstrated that small-molecule modulators of endomembrane channels can be used to tune organelle function and trafficking. The diseases associated with IP3Rs and RyRs have been extensively reviewed previously (161, 386–389), so below we focus on the roles of lysosomal channels in diseases.

### 5.1. TRPML1 in ML-IV and LSDs

Type IV mucolipidosis (ML-IV) is a rare autosomal recessive LSD and a neurodevelopmental disorder that is clinically manifested by psychomotor retardation, retinal degeneration, intellectual disability, and achlorhydria (215, 216, 390, 391) (TABLE 2). More than 30 loss-of-function mutations in *TRPML1* were identified as the underlying causes of ML-IV (215–217, 392). In addition, several LSDs are caused by the mutations in the genes that regulate the production of the signaling cues that directly or indirectly influence TRPML1’s channel function; those LSDs manifest ML-IV-like phenotypes due to lysosomal trafficking defects (62, 239). For instance, excessive sphingomyelin accumulation causes a pathological inhibition of TRPML1 in Niemann–Pick disease type C (NPC) (62). Likewise, mutations in PIKfyve, the PI(3,5)P_2_-synthesizing enzyme, and in OCRL, a PI(4,5)P_2_ phosphatase, also cause lysosomal storage and ML-IV-like symptoms (120, 233). In addition, lysosomal adenosine accumulation may inhibit TRPML1 to cause LSD-like phenotypes in ADA KO cells (238). Given the similarities between LSDs and common neurodegenerative diseases such as AD and PD (393–395), it is not surprising that TRPML1 signaling is also impaired in AD and PD (63, 64, 396). Although no massive Aβ/Tau accumulation has been reported in TRPML1 KO mice or ML-IV patients, at least in young animals and patients, TRPML1 is reportedly implicated in AD pathogenesis in multiple studies (63, 213, 390, 396).

### 5.2. TRPML1 and Other Lysosomal Channels as Drug Targets for LSDs, AD, and PD

Mutations in hydrolases or catabolite exporters also cause lysosomal storage, which in turn affects lysosomal degradation and trafficking to cause secondary storage, resulting in a vicious cycle (25). TRPML1 is a central regulator of lysosomal trafficking, and compromised TRPML1 activity may act as a common pathogenic mechanism for many LSDs (13, 62, 238, 244, 397). Therefore, deinhibition of TRPML1 could break the vicious cycle, and activation of TRPML1 may facilitate lysosomal trafficking to clear lysosomal storage. In patients with ML-IV caused by hypofunctional *TRPML1* mutations, small-molecule agonists of TRPML1 were shown to restore normal lysosomal trafficking functions (244). Furthermore, TRPML1 overexpression and small-molecule TRPML1 agonists can increase cholesterol clearance in NPC cells (62, 269). It is worth noting that TRPML1 activation may also be able to boost phagocytic clearance of apoptotic debris in the brain (88, 391). Hence, manipulating the expression and activity of TRPML1 and other lysosomal channels may provide an exciting opportunity to clear lysosomal storage in the cells and animal models of LSD. As lysosomal trafficking defects are commonly seen in many LSDs (398), this approach could potentially provide a novel therapeutic approach to treat many other LSDs. Given the functional interactions between Lyso-BK and TRPML1, Lyso-BK agonists were shown to display similar storage-reducing effects (239). Moreover, given that TPCs also regulate many lysosomal trafficking steps like TRPML1 does (43), TPC agonists were also shown to be effective in cellular clearance for multiple LSDs (397). Hence, although we focus our discussions on TRPML1, the targeting strategies could be extended to other lysosomal channels for other LSDs as well.

If increasing TRPML1 expression/activity promotes cellular clearance, cellular conditions or manipulations that can boost TRPML1 expression or activity may also enhance lysosome function. TFEB is a master regulator of lysosomal biogenesis and autophagy (59, 208, 399). When lysosomes are under conditions of stress, TFEB proteins translocate from the cytosol to the nucleus, thereby inducing the expression of hundreds of autophagy- and lysosome-related genes (59, 399). In multiple sulfatase deficiency (MSD) and mucopolysaccharidosis type IIIA (MPS-IIIA), two glycosaminoglycan (GAG) storage LSDs, TFEB overexpression was sufficient to reduce lysosomal GAG accumulation (255). Similarly, TFEB-stimulated cellular clearance may be effective in the mouse models of Batten disease, neuronal ceroid lipofuscinoses (NCLs), and Pompe disease (255, 400). Strikingly, the beneficial effects of TFEB on various LSDs are dependent on TRPML1 and lysosomal exocytosis (255, 400). Considering that TRPML1 is upregulated by TFEB overexpression (401), the TFEB-TRPML1 interaction may play a pivotal role in promoting lysosomal exocytosis and trafficking for cellular clearance. Small-molecule agonists of TRPML1 were shown to reduce Aβ and α-synuclein accumulation in cellular models of AD and PD, respectively (63, 64, 396). Hence, TRPML1 may represent a general therapeutic target for LSDs, AD, and PD, as well as other age-related metabolic and neurodegenerative diseases. Although an in vivo efficacy of the synthetic agonists of TRPML1 has not been reported for any central nervous system (CNS) diseases, ML-SA compounds were shown to be muscle protective in a mouse model of DMD, a non-CNS disease (63, 243).

### 5.3. TMEM175 in PD

Recent GWAS studies identified TMEM175 as a novel genetic risk factor for PD (339, 341, 342, 402, 403) (TABLE 2). For instance, the p.M393T variant of TMEM175 is more prevalent in the PD cohorts (404), and M393T in human TMEM175 was found to be a hypofunctional mutation in whole LEL recordings (104, 344). The autophagy-lysosome system and hydrolytic enzymes, such as cathepsin B and cathepsin D in lysosomes, play a critical role in α-synuclein degradation (356, 394, 405–410). Hypoactivity of TMEM175/LyPAP, as seen in TMEM175 KO and M393T knockin mice, may be responsible for the increased aggregation of α-synuclein in the brain (104, 205, 343, 344). In a pharmacologically induced PD model, however, TMEM175 KO was found to mitigate motor impairment and dopaminergic neuron loss (350). Hence, TMEM175 may contribute to PD pathology differently in the different stages of PD. Before diagnosis, PD patients may already exhibit typical symptoms of α-synucleinopathies such as rapid eye movement (REM) sleep behavior disorders. Notably, *TMEM175* is also a risk gene for sleep disorders (404). It remains to be tested whether TMEM175 agonists, with appropriate dosing, may compensate for the lysosomal deficiencies and PD pathology caused by loss-of-function mutations of TMEM175. Finally, as defective lysosomal acidification is implicated in many LSDs and neurogenerative diseases beyond PD (358), TMEM175 may also represent a general target for lysosomal diseases.

## 6. SUMMARY AND FUTURE DIRECTIONS

Endomembrane organellar channels play central roles in signal transduction, macromolecular synthesis, macromolecular degradation, membrane trafficking, and maintenance of organelle homeostasis. However, the molecular identities and functional roles of many endogenous organellar channels are still not clear. About two dozen endomembrane organellar channels have now been identified and characterized, many with organellar electrophysiology. Among the most extensively studied are several ER and lysosomal channels including IP3Rs and TRPMLs. With the development of various channel-based assays, cellular cues that serve as endogenous agonists, as well as small-molecule modulators, were identified. With the use of various pharmacological tools and genetic models, some organellar functions of several identified endomembrane channels have been defined. However, it remains largely unexplored how most organellar channels are regulated by environmental factors and cellular cues. To unambiguously define organellar channels of endomembrane channels, high-resolution live imaging is necessary to detect organelle dynamics under various physiological conditions and upon acute manipulation of the activity of endomembrane channels. Although molecular expression analyses and lipid bilayer studies have aided in revealing several candidate organellar channels, most organellar channels still need to be studied in their native settings with organellar patch-clamp and organellar ion imaging techniques. In the future, we will likely see more organellar channels molecularly identified. More specifically, chemical agonists and inhibitors of organellar channels will be developed, which in combination with the genetic tools, e.g., CRISPR-Cas9, may help precisely define the physiological functions of each organellar channel. As organellar channels are often regulated by more than one cellular cue, it is necessary to perform studies with site-specific mutational knockin models to define the specific functions of each activating cue. Finally, proof-of-concept studies have established the therapeutic potential of endomembrane channels. The central concept that enhancing lysosomal trafficking may be able to alleviate the pathological symptoms in most LSDs regardless of the primary deficiency is appealing. Whether endolysosomal channels can be common targets for the treatments of many LSDs and AD/PD still awaits more in vitro cellular and in vivo animal studies.

## GRANTS

The work in the authors’ laboratory is supported by grants from the National Key R&D Program of China (no. 2022YFE0210100) and the National Science Foundation of China (NSFC, no. 92354306), start-up funds from the Liangzhu Laboratory and the Zhejiang University, and the New Cornerstone Investigator Program.

## DISCLOSURES

H.X. is a scientific cofounder and a partial owner of Lysoway Therapeutics Inc (Boston, MA). None of the other authors has any conflicts of interest, financial or otherwise, to disclose.

## AUTHOR CONTRIBUTIONS

H.X. conceived and designed research; M.H. and H.X. interpreted results of experiments; M.H., S.L., F.H., and H.X. prepared figures; M.H., X.F., Q.L., and H.X. drafted manuscript; M.H., X.F., Q.L., and H.X. edited and revised manuscript; H.X. approved final version of manuscript.
